# Integrated Multi-Omics Analysis Reveals Complex Cytotoxicity-Associated Molecular Response Patterns of Representative Toxins from Four Classes of Lipophilic Algal Toxins in Neuro-2a Cells

**DOI:** 10.3390/toxins18070274

**Published:** 2026-06-23

**Authors:** Xueru Wei, Pengrui Ren, Junkai Feng, Jingyuan Shi, Peipei Zhang, Hongjun Li

**Affiliations:** 1 College of Marine Science Technology and Environment, Dalian Ocean University, Dalian 116023, China; weixueru2002@163.com; 2 State Environmental Protection Key Laboratory of Coastal Ecosystem, National Marine Environmental Monitoring Center, Dalian 116023, China; fengjk6960@163.com (J.F.); jyshi@nmemc.org.cn (J.S.); hjli@nmemc.org.cn (H.L.); 3 Key Laboratory of Industrial Ecology and Environmental Engineering (Ministry of Education), School of Environmental Science and Technology, Dalian University of Technology, Dalian 116024, China; rpr02200059@163.com

**Keywords:** lipophilic marine toxins, Neuro-2a cells, cytotoxicity, multi-omics, molecular responses

## Abstract

Lipophilic marine toxins (LMTs) are important toxic risk factors in marine ecosystems and seafood safety, yet the comparative cytotoxicity-associated molecular responses of different LMT classes remain unclear. Here, Neuro-2a cells were exposed to four representative LMTs—dinophysistoxin-1 (DTX1), azaspiracid-3 (AZA3), yessotoxin (YTX), and pectenotoxin-2 (PTX2)—and acute cytotoxicity was evaluated together with integrated transcriptomic, proteomic, and metabolomic analyses. Cell viability assays showed a cytotoxic potency order of DTX1 > AZA3 > YTX > PTX2. Integrated multi-omics analysis revealed that DTX1, the most cytotoxic toxin, caused the broadest molecular perturbations, mainly involving mitochondrial energy metabolism, p53-mediated stress responses, and multilayered metabolic networks. AZA3 and YTX induced intermediate cytotoxicity and showed partially similar perturbation patterns, particularly affecting cytoskeleton-related, immune-related, and metabolism-related processes. In contrast, PTX2, the least cytotoxic toxin, produced more limited responses mainly involving tyrosine metabolism and the cGMP–PKG signaling network. Overall, molecular perturbation patterns generally corresponded to acute cytotoxic potencies, while each toxin exhibited distinct key pathways and functional modules. These findings provide a multi-omics basis for cytotoxic responses of representative LMT classes and guide subsequent functional validation.

## 1. Introduction

Over the past few decades, environmental factors such as global warming and the intensification of eutrophication in coastal waters have led to an increased frequency and expanding scale of harmful algal blooms, thereby drawing widespread attention to marine algal toxins [1,2]. More than 300 marine algal toxins have been identified to date, of which approximately 90% are classified as lipophilic toxins [3,4]. Lipophilic marine toxins (LMTs) are a group of toxic secondary metabolites characterized by structurally stable chemical properties and resistance to degradation [5]. Based on their core chemical structures, LMTs are mainly classified into the okadaic acid group, azaspiracid group, yessotoxin group, and pectenotoxin group, among others [4]. Among them, dinophysistoxin-1 (DTX1), azaspiracid-3 (AZA3), yessotoxin (YTX), and pectenotoxin-2 (PTX2) are representative congeners with relatively high toxicity within their respective groups [6]. Beyond toxicity, DTX1, AZA3, YTX, and PTX2 are also relevant to environmental occurrence, recurrent shellfish contamination, and seafood safety monitoring [7,8]. LMTs can not only undergo transport and transformation within marine ecosystems, but also readily accumulate over the long term in marine sediments and in organisms such as shellfish [9]. Such accumulation facilitates their transfer through the food chain and ultimately poses a direct threat to human health through seafood consumption [10].

Previous studies have reported that different LMTs can affect several cellular processes, including cytoskeletal disruption, dysregulation of signaling pathways, induction of oxidative stress, activation of apoptosis, and impairment of cell membrane function, among others. Specifically, dinophysistoxin-1 (DTX1), a typical diarrhetic shellfish toxin, exerts its toxicity mainly by inhibiting the activities of protein phosphatase 1 (PP1) and protein phosphatase 2A (PP2A), thereby disrupting the balance of intracellular signaling and subsequently inducing toxic responses such as cytoskeletal rearrangement and tight junction disassembly. This mechanism has been validated primarily in epithelial cell models such as HEp-2 and Caco-2 cells [11,12]. Azaspiracid-3 (AZA3) has been reported to affect intracellular second-messenger systems and can induce cytotoxicity and cytoskeletal disruption by modulating intracellular calcium levels, cyclic adenosine monophosphate (cAMP) levels, and low-density lipoprotein receptor (LDLR) expression. Current mechanistic studies have mainly relied on HepG2 hepatocytes and Jurkat T lymphocytes [13,14]. In contrast, yessotoxin (YTX) has been associated with cellular calcium transport across membranes and triggering caspase-mediated apoptotic pathways. Its toxicity is characterized by marked dose dependence and delayed onset, and HeLa cells have been widely used as the principal model for mechanistic investigations [15,16]. The molecular mechanism of pectenotoxin-2 (PTX2) has been less extensively studied. Nevertheless, related toxins have been reported to affect cell adhesion by affecting the integrity of cytoskeletal components such as microfilaments and microtubules, with most available evidence derived from ex vivo experiments using shellfish tissues or plankton extracts [17,18]. However, most existing studies have focused on individual toxins, making it difficult to directly compare the relative cytotoxic potency of different LMTs and their associated molecular response patterns. The neuroblastoma cell line Neuro-2a has been reported to be sensitive to a variety of LMTs and, because of its operational simplicity and good reproducibility, has become a preferred in vitro model for marine algal toxin toxicity screening [19,20]. Therefore, in the present study, Neuro-2a cells were selected as an in vitro model to compare the cytotoxic effects and molecular responses induced by these four toxins under standardized experimental conditions. Although Neuro-2a cells provide a reproducible and convenient model for comparative toxicity screening, they cannot fully recapitulate the complexity of primary neuronal cultures or in vivo systems, including mature neuronal networks, cell–cell interactions, and organism-level toxicokinetic responses [21].

Integrated multi-omics analysis, which combines transcriptomics, proteomics, and metabolomics, enables the systematic characterization of toxicity-associated molecular responses across multiple levels, including gene expression, protein function, and metabolic perturbations, and has become an important paradigm in modern toxicology [22]. However, in the field of LMTs, studies on cytotoxicity-associated molecular responses based on integrated multi-omics approaches remain relatively limited, and the similarities and differences among LMT-induced alterations at the transcriptomic, proteomic, and metabolomic levels therefore remain unclear. The present study aimed to compare the acute cytotoxicity-associated molecular responses induced by different classes of LMTs, including the OA, AZA, YTX, and PTX groups, in Neuro-2a cells. Specifically, representative congeners exhibiting the highest toxicity toward Neuro-2a cells within these four LMT groups, namely DTX1, AZA3, YTX, and PTX2, were selected [6], and their cytotoxic responses in Neuro-2a cells were investigated. Using transcriptome-enriched pathways as the primary framework, and integrating key molecular changes identified in the proteome and metabolome, a multi-omics strategy was employed to compare the common and specific molecular responses induced by different toxins at the levels of gene expression, protein function, and metabolic networks. The results provide a multi-omics basis for comparing cytotoxicity-associated molecular responses induced by different classes of LMTs and lay the foundation for subsequent functional validation.

## 2. Results

### 2.1. Cytotoxic Effects of Four LMTs on Neuro-2a Cells

**Figure 1:**
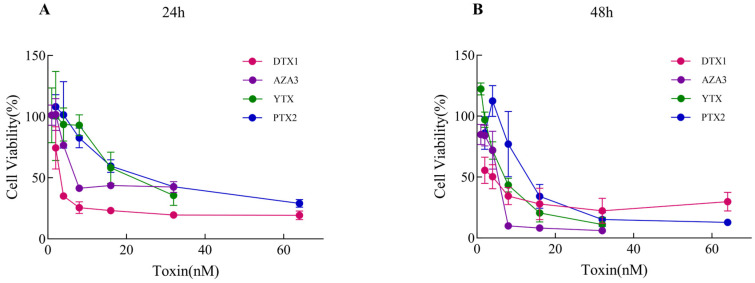
Effects of dinophysistoxin-1 (DTX1), azaspiracid-3 (AZA3), yessotoxin (YTX), and pectenotoxin-2 (PTX2) on Neuro-2a cell viability. (**A**) Cells were treated with the indicated concentrations of DTX1, AZA3, YTX and PTX2 for 24 h. (**B**) Cells were treated with the indicated concentrations of DTX1, AZA3, YTX and PTX2 for 48 h. The results were presented as the percent cell viability compared with the untreated control group. Data are the means ± SD (*n* = 3).

To evaluate the effects of DTX1, AZA3, YTX, and PTX2 on the viability of Neuro-2a cells, cell survival was determined by the 3-(4,5-dimethylthiazol-2-yl)-2,5-diphenyltetrazolium bromide (MTT) assay after 24 and 48 h of exposure to different toxin concentrations. As shown in Figure 1 (with fitted dose–response curves provided in Figure S1), all four LMTs reduced the viability of Neuro-2a cells in a time- and dose-dependent manner. Based on the calculated half-maximal inhibitory concentration (IC_50_) values (Table 1), the cytotoxic potencies of the four LMTs differed significantly (*p* < 0.05). After 24 h of exposure, their toxicity toward Neuro-2a cells decreased in the following order: DTX1 (IC_50_ = 3.39 nM) > AZA3 (9.67 nM) > YTX (21.88 nM) > PTX2 (26.77 nM). When the exposure time was extended to 48 h, the IC_50_ values of all toxins decreased, and their cytotoxicity decreased in the following order: DTX1 (2.62 nM) > AZA3 (4.32 nM) > YTX (7.45 nM) > PTX2 (10.55 nM).

### 2.2. Transcriptomic Responses Induced by Four LMTs

**Figure 2:**
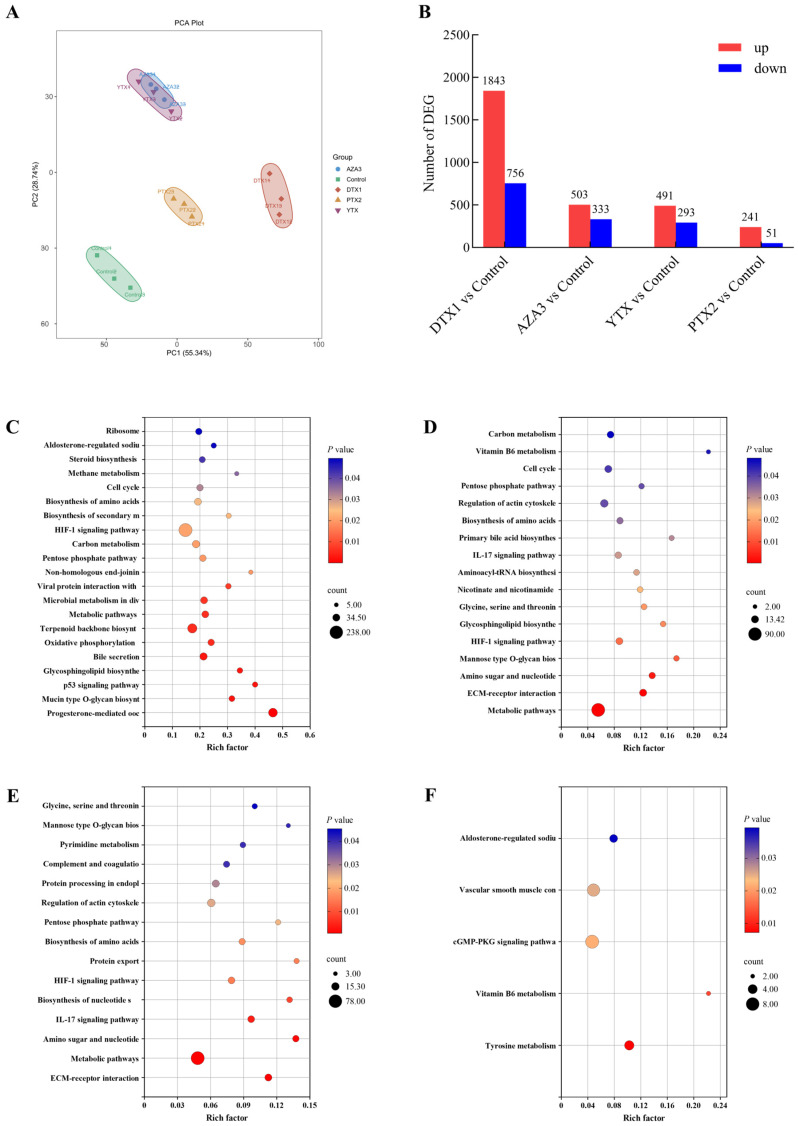
Kyoto Encyclopedia of Genes and Genomes (KEGG) pathway enrichment analysis of differentially expressed genes (DEGs) in Neuro-2a cells exposed to DTX1, AZA3, YTX, and PTX2. (**A**) Principal component analysis (PCA) of transcriptomic profiles. PCA was performed on the normalized expression data of all samples. Each dot represents a biological replicate (*n* = 3), with colors indicating different treatment groups. Ellipses represent the 95% confidence regions for the corresponding groups. Percentages on the axes represent the variance explained by PC1 and PC2. The tight clustering of control samples demonstrates high reproducibility. (**B**) Landscape of differentially expressed genes (DEGs) induced by each toxin. Stacked bar plot showing the number of up- and down-regulated DEGs for each comparison [false discovery rate (FDR) ≤ 0.05 and |log_2_ fold change (log_2_FC)| > 1]. (**C**–**F**) KEGG pathway enrichment analysis of DEGs across four toxin treatments. Bubble plots showing the top enriched KEGG pathways for DTX1 vs. Control (**C**), AZA3 vs. Control (**D**), YTX vs. Control (**E**), and PTX2 vs. Control (**F**). Bubble size indicates the number of DEGs mapped to each pathway, and bubble color represents enrichment significance (−log10 *p*-value). Rich factor is defined as the ratio of DEGs mapped to a pathway to the total number of annotated genes in that pathway.

Principal component analysis (PCA) based on the normalized expression matrix showed a clear separation between all toxin-treated groups and the control group (Figure 2A). Biological replicates within each group clustered closely within their corresponding confidence ellipses, indicating good within-group consistency and reproducibility of the transcriptomic dataset. The AZA3 and YTX groups were positioned relatively close to each other in the PCA space, suggesting that these two toxins may elicit similar transcriptional regulatory patterns. In contrast, the DTX1 group was clearly separated from the control group, whereas the PTX2 group was located relatively closer to the control, indicating that the transcriptional responses induced by the different toxins were distinct.

As shown by the numbers of differentially expressed genes (DEGs) in Figure 2B, the DTX1 group exhibited the largest number of DEGs, with 2599 altered genes, including 1843 upregulated and 756 downregulated genes. The AZA3 and YTX groups showed fewer DEGs, with 836 (503 upregulated and 333 downregulated) and 784 (491 upregulated and 293 downregulated) genes, respectively, and their ratios of upregulated to downregulated genes were relatively similar. The PTX2 group exhibited the smallest number of DEGs, with only 292 altered genes, including 241 upregulated and 51 downregulated genes.

The significantly enriched Kyoto Encyclopedia of Genes and Genomes (KEGG) pathways in the four LMT-treated groups are shown in Figure 2C–F. Overall, DEGs identified in the DTX1, AZA3, and YTX groups were enriched in metabolism-related pathways. Among them, the DTX1 group was uniquely enriched in oxidative phosphorylation, p53 signaling pathway, biosynthesis of secondary metabolites, and microbial metabolism in diverse environments. In addition to metabolism-related pathways, DEGs in the AZA3 and YTX groups were enriched in IL-17 signaling pathway, regulation of actin cytoskeleton, and ECM–receptor interaction. DEGs in the AZA3 group were uniquely enriched in primary bile acid biosynthesis and glycosphingolipid biosynthesis–lacto and neolacto series, whereas DEGs in the YTX group were uniquely enriched in protein export and biosynthesis of nucleotide sugars. In contrast, DEGs in the PTX2 group exhibited fewer enriched pathways and were uniquely enriched in tyrosine metabolism and cGMP–PKG signaling pathway.

### 2.3. Proteomic and Metabolomic Responses to Four LMTs

To further support the key enriched pathways identified in the transcriptomic analysis, proteomic and metabolomic analyses were performed to investigate toxin-induced changes at the protein and metabolite levels. In the proteomic analysis, the DTX1, AZA3, and YTX groups were enriched in pathways related to basal metabolism, immune response, and calcium signaling, respectively, whereas no significantly enriched pathways were identified in the PTX2 group (Figure S2). In the metabolomic analysis, the DTX1 group was mainly enriched in pathways related to cholesterol metabolism and vitamin digestion and absorption, whereas the AZA3 and YTX groups were primarily associated with energy metabolism, such as oxidative phosphorylation. In contrast, the metabolic alterations in the PTX2 group were relatively limited and were mainly associated with amino acid metabolism (Figure S3).

### 2.4. Integrated Multi-Omics Analysis of Key Enriched Pathways

**Figure 3:**
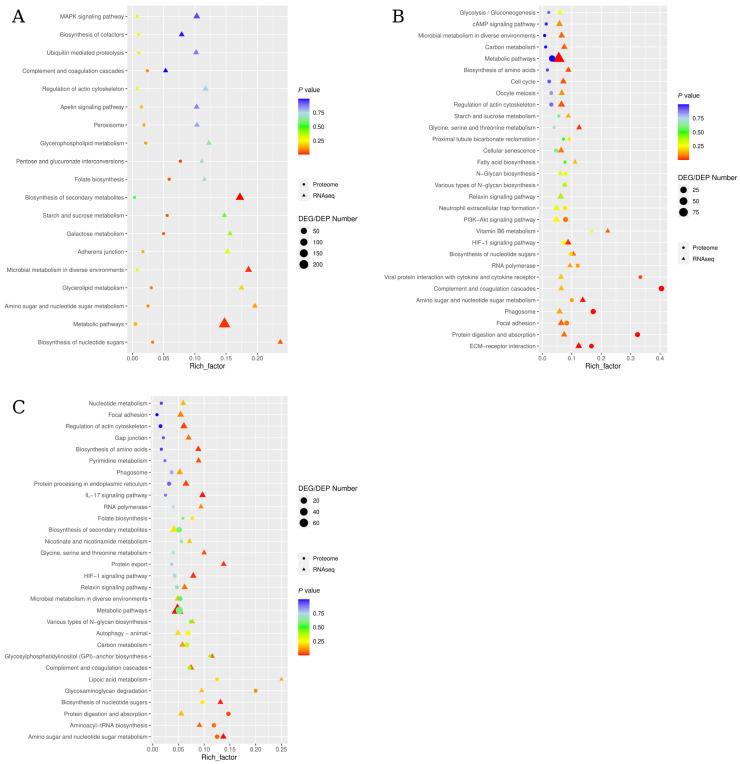
KEGG pathways shared between transcriptomic and proteomic datasets. (**A**) DTX1-treated group; (**B**) AZA3-treated group; (**C**) YTX-treated group. Triangles and circles represent pathways enriched in the transcriptomic and proteomic datasets, respectively. Bubble size indicates the number of DEGs or DEPs mapped to each pathway, and bubble color represents enrichment significance (−log10 *p*-value). Rich factor represents the ratio of DEGs or differentially expressed proteins (DEPs) mapped to a pathway to the total number of annotated genes or proteins in that pathway.

**Figure 4:**
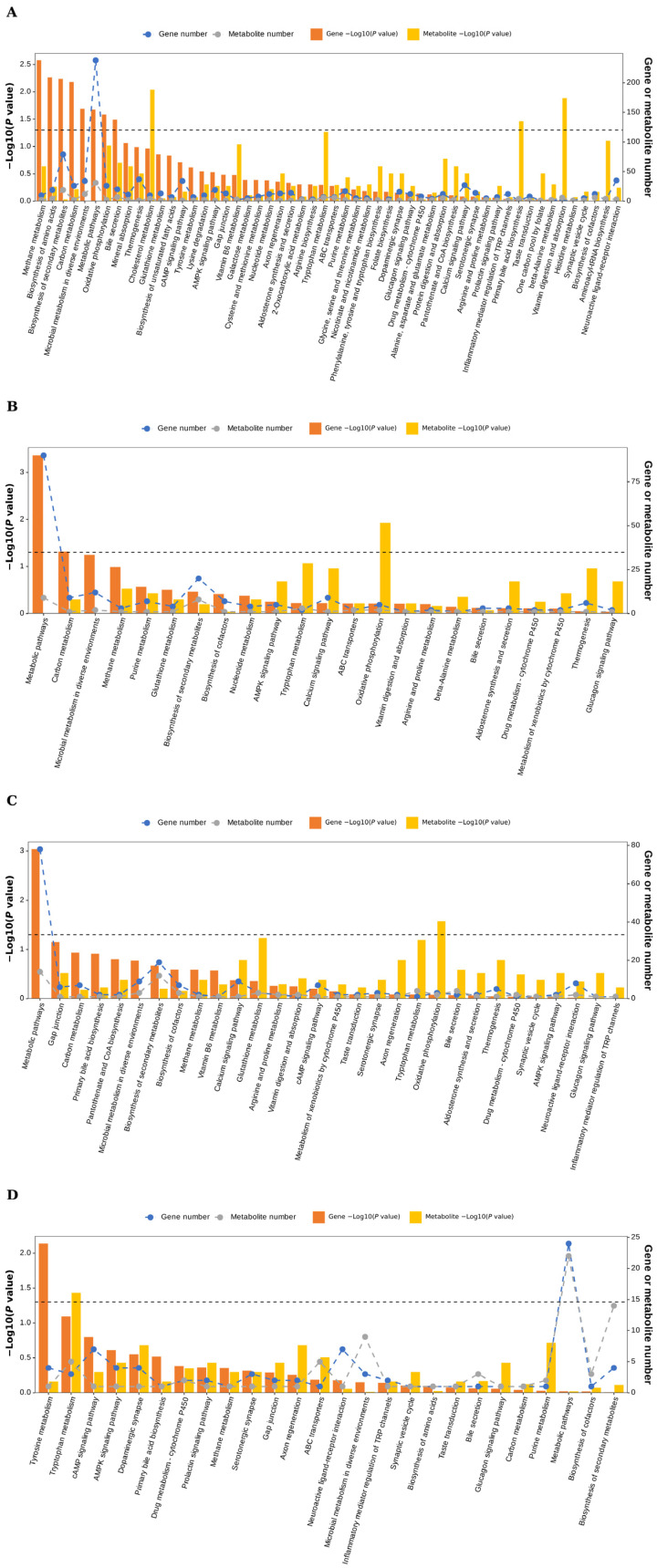
KEGG pathways shared between transcriptomic and metabolomic datasets. (**A**) DTX1-treated group; (**B**) AZA3-treated group; (**C**) YTX-treated group; (**D**) PTX2-treated group. Orange and yellow bars represent the enrichment significance (−log10 *p*-value) of genes and metabolites, respectively. Blue and gray dashed lines indicate the number of genes and metabolites associated with each pathway. The horizontal dashed line represents the significance threshold (*p* = 0.05).

Based on the key enriched pathways identified in the transcriptomic analysis, we further searched for the corresponding differential molecules in the proteomic and metabolomic datasets and summarized the DEGs, differentially expressed proteins (DEPs), and differentially expressed metabolites (DEMs) involved in each pathway (Table S2). Using the differential molecules compiled in Table S2 as the basis for cross-omics comparison, we identified shared enriched pathways at both the transcriptomic–proteomic and transcriptomic–metabolomic levels, as shown in Figure 3 and Figure 4. Overall, only a limited number of pathways contained differential molecules shared between the transcriptome and proteome or between the transcriptome and metabolome. Representative pathways containing differential molecules across multiple omics layers were further subjected to Spearman correlation analysis. These correlation analyses were exploratory and were used to identify candidate cross-omics associations rather than direct regulatory or causal relationships.

**Figure 5:**
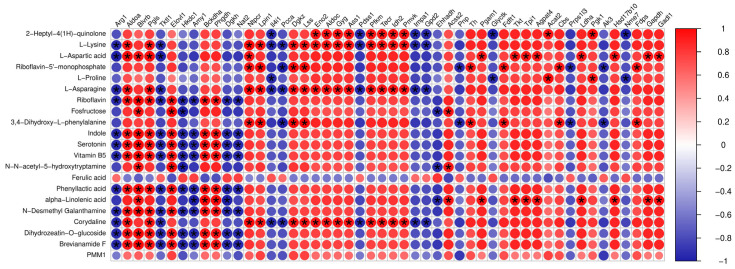
Spearman correlation heatmap of all DEGs with DEPs or differentially expressed metabolites (DEMs) in biosynthesis of secondary metabolites (DTX1-treated Neuro-2a cells). Red and blue colors indicate positive and negative correlations, respectively. For clarity, the heatmap shows the top 50 genes with the most significant FDR-adjusted correlations, and the complete heatmap is provided in the Supplementary Materials (Figure S4). PMM1, phosphomannomutase 1 (* FDR-adjusted *p* < 0.05).

**Figure 6:**
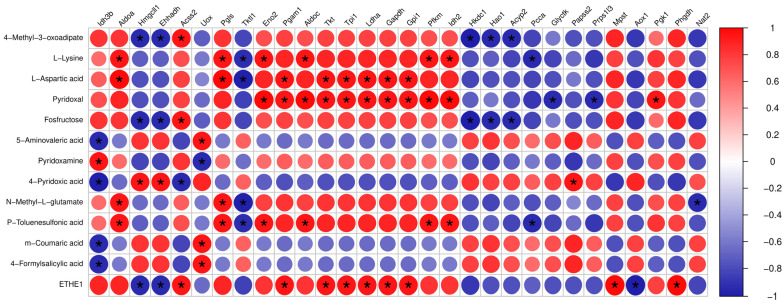
Spearman correlation heatmap of all DEGs with DEPs or DEMs in Microbial metabolism in diverse environments (DTX1-treated Neuro-2a cells). Red and blue colors indicate positive and negative correlations, respectively. For clarity, the heatmap shows the top 30 genes with the most significant FDR-adjusted correlations, and the complete heatmap is provided in the Supplementary Materials (Figure S5). ETHE1, persulfide dioxygenase ETHE1, mitochondrial (* FDR-adjusted *p* < 0.05).

The results showed that the key multi-omics alterations identified in the DTX1 group were mainly associated with energy metabolism, stress response, and broad metabolic networks. Among these, differential metabolites were also identified in the oxidative phosphorylation pathway, and *mt-Co1*, *Cox4i2*, *Ndufa4l2*, and *Atp5d* were positively correlated with the metabolites riboflavin-5′-monophosphate (FMN) and nicotinamide adenine dinucleotide (reduced) (NADH) (Table S3). In addition, biosynthesis of secondary metabolites and microbial metabolism in diverse environments were among the few pathways in this study that contained differential molecules across all three omics layers. In biosynthesis of secondary metabolites, *Tktl1* was significantly negatively correlated with L-aspartic acid, L-lysine, and riboflavin (Figure 5). In microbial metabolism in diverse environments, the persulfide dioxygenase ETHE1, mitochondrial, was negatively correlated with *Hkdc1*, *Tktl1*, and *Ehhadh*, among which the negative correlation with Ehhadh was significant. The metabolites L-aspartic acid, fosfructose, pyridoxamine, and pyridoxal also showed overall negative correlations with the above genes, among which the negative correlation of *Hkdc1* with fosfructose was statistically significant (Figure 6).

The key multi-omics alterations identified in the AZA3 group were mainly concentrated in processes related to immune inflammation, cytoskeletal regulation, and lipid metabolism. In regulation of actin cytoskeleton, Itgb4, Itga1, Vcl, and Fn1 showed overall positive correlations between the transcriptomic and proteomic datasets, with selected correlations reaching statistical significance (Table S4). In ECM-receptor interaction, integrin- and collagen-related genes showed significant positive correlations across the two omics layers (Table S5).

The key multi-omics alterations identified in the YTX group were mainly associated with immune inflammation, cytoskeletal regulation, and protein processing. In IL-17 signaling pathway, *Fosb* was significantly positively correlated with the proteomic molecule TANK-binding kinase 1 (TBK1), whereas Rela showed a negative correlation trend with TBK1. In regulation of actin cytoskeleton, the protein IQGAP1 was significantly positively correlated with the genes Itga1 and Vcl. In protein export, Hspa5 showed a positive correlation trend with proteomic molecules. In biosynthesis of nucleotide sugars, *Nagk* was significantly positively correlated with UAP1L1 and HK2, while showing negative correlations with some proteomic molecules (Table S6).

In contrast, the PTX2 group showed a relatively limited range of multi-omics alterations, mainly involving amino acid metabolism and the cGMP–PKG signaling pathway. Among these, tyrosine metabolism contained differential molecules at both the transcriptomic and metabolomic levels, and 3,4-dihydroxy-L-phenylalanine (DOPA) was negatively correlated with Aox4 (Figure S6).

### 2.5. Reverse Transcription Quantitative Polymerase Chain Reaction Validation of Representative Transcriptomic Genes

**Figure 7:**
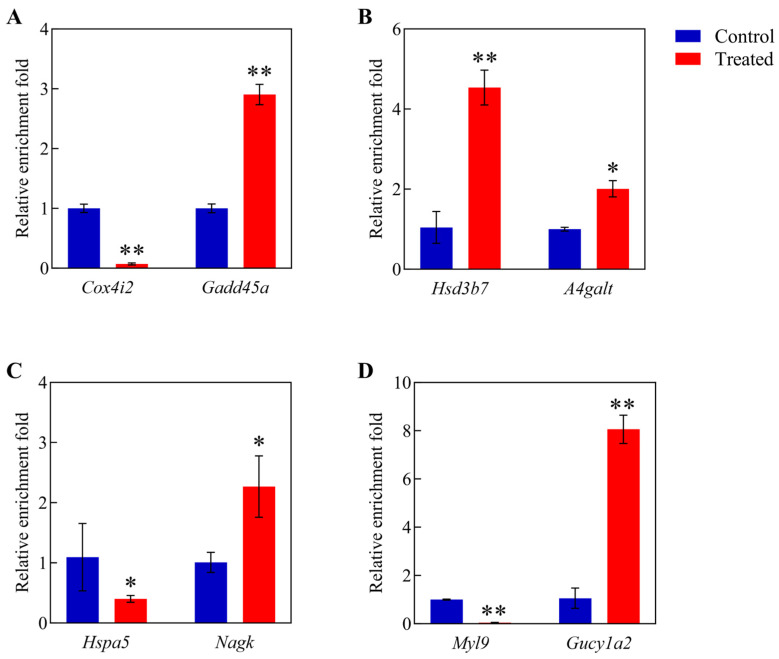
Reverse transcription quantitative polymerase chain reaction (RT-qPCR) validation of representative differentially expressed genes identified by transcriptomic analysis. Neuro-2a cells were treated with DTX1, AZA3, YTX or PTX2 at the indicated concentrations for 48 h. (**A**) DTX1-treated group; (**B**) AZA3-treated group; (**C**) YTX-treated group; (**D**) PTX2-treated group. The mRNA levels of the indicated genes were measured by RT-qPCR and normalized to *β-actin* using the 2^−ΔΔCt^ method, with the control group set as the calibrator (mean ΔCt = 0). Data are presented as mean ± SD (*n* = 3). Statistical significance was determined by independent two-tailed Student’s *t*-test. * *p* < 0.05, ** *p* < 0.01 compared with the control group.

To validate the reliability of the transcriptomic data, eight representative DEGs (*Cox4i2*, *Gadd45a*, *Hsd3b7*, *A4galt*, *Hspa5*, *Nagk*, *Myl9*, and *Gucy1a2*) were selected for reverse transcription quantitative polymerase chain reaction (RT-qPCR) analysis. As shown in Figure 7, compared with the control group, the relative expression patterns of these genes after the corresponding toxin treatments were fully consistent with the transcriptomic sequencing results. *Cox4i2*, *Hspa5*, and *Myl9* were significantly downregulated, whereas *Gadd45a*, *Hsd3b7*, *A4galt*, *Nagk*, and *Gucy1a2* were significantly upregulated (*p* < 0.05). These RT-qPCR results, based on eight representative DEGs, support the reliability of the RNA-seq data for selected transcriptional responses, but do not validate downstream pathway-level mechanisms, which require further functional verification.

## 3. Discussion

In the present study, we compared the acute cytotoxicity of four representative LMTs in Neuro-2a cells and characterized their molecular response patterns using a multi-omics approach. The results revealed marked differences in both cytotoxic potency and molecular perturbation patterns among the toxins, thereby providing multi-level evidence for understanding cytotoxicity-associated molecular responses induced by LMTs in Neuro-2a cells.

### 3.1. Distinct Cytotoxic Potencies and Toxin-Specific Molecular Perturbation Patterns

The MTT results showed that all four toxins exerted time- and dose-dependent cytotoxic effects on Neuro-2a cells, with the order of cytotoxic potency at 48 h being DTX1 > AZA3 > YTX > PTX2. Transcriptomic analysis further demonstrated that the molecular responses induced by the different toxins were markedly distinct. PCA showed that the DTX1 group was separated furthest from the control group, whereas the PTX2 group was the closest, while the AZA3 and YTX groups were positioned relatively close to each other in the PCA space, suggesting that these two toxins may share similar transcriptional regulatory patterns. The numbers of DEGs were consistent with the PCA results: the DTX1 group showed the largest number of DEGs, the AZA3 and YTX groups showed intermediate numbers, and the PTX2 group showed the fewest. These findings indicate that even at approximately equitoxic concentrations, the transcriptional responses induced by the different toxins differed substantially. KEGG pathway enrichment analysis further showed that DEGs associated with the different toxin treatments was enriched in distinct functional pathways, indicating differentiated molecular response patterns and suggesting potential biological processes associated with their different cytotoxic responses. Several transcriptome-level findings were supported by corresponding protein or metabolite changes, whereas others remained specific to individual omics layers.

As the most cytotoxic toxin examined in this study, DTX1 was primarily associated with alterations in pathways related to mitochondrial energy metabolism and the p53-centered stress response, with these responses further extending to carbohydrate and amino acid metabolic networks. Within oxidative phosphorylation, the transcriptomic changes were accompanied by corresponding alterations at the metabolite level. DTX1 treatment downregulated multiple genes encoding subunits of the mitochondrial respiratory chain complexes, including *mt-Co1*, *Cox4i2*, *Ndufa4l2*, and *Atp5d*. At the metabolomic level, the levels of NADH, a key electron donor in the electron transport chain, and FMN, a core cofactor of complex I, were also significantly reduced. The positive correlations between these genes and metabolites further connected the transcriptomic and metabolomic responses within oxidative phosphorylation. These coordinated transcriptomic and metabolomic changes suggest that DTX1 exposure is associated with altered mitochondrial energy metabolism. Mitochondrial impairment is commonly implicated in cellular responses to many toxicants [23], whereas DTX1 is known mainly to interfere with cellular signaling through inhibition of PP1 and PP2A [11]. Thus, the present findings provide biological context linking DTX1 exposure to mitochondrial energy-related molecular responses in Neuro-2a cells. In p53 signaling pathway, DTX1 upregulated pro-apoptotic genes (*Apaf1* and *Pidd1*) and negative regulators of the cell cycle (*Gadd45a* and *Ccng1*), while downregulating cell cycle-promoting genes (*Ccnb1*, *Cdk1*, and *Rrm2*), suggesting an association with p53-mediated stress responses and cell-cycle-related alterations. This is consistent with previous reports showing that OA, another member of the okadaic acid group, upregulates p53 and induces apoptosis through inhibition of PP1/PP2A [24], and that DTX1 exposure causes cell cycle arrest accompanied by elevated ROS levels [25]. Additional studies in neuroblastoma models have also shown that okadaic acid-group toxins can induce cytotoxicity and apoptotic changes in neuronal cell contexts [20,26]. Together, these findings suggest that okadaic acid-group toxins may be associated with cell-cycle alterations involving the p53 signaling pathway.

Beyond energy metabolism and the stress-response core, DTX1-associated molecular alterations were also observed in carbohydrate and amino acid metabolic networks. Notably, microbial metabolism in diverse environments and biosynthesis of secondary metabolites were the only two pathways in which differential molecules were detected across all three omics layers, providing cross-omics support for DTX1-associated metabolic alterations. In microbial metabolism in diverse environments, genes encoding key glycolytic enzymes were downregulated, whereas genes involved in alternative metabolic routes, including *Hkdc1*, *Glyctk*, and *Tktl1*, were upregulated. These transcriptomic changes were accompanied by increased ETHE1 abundance at the protein level and decreased levels of L-aspartic acid, fosfructose, and pyridoxamine were observed. Correlation analysis further showed that ETHE1 was significantly negatively correlated with *Hkdc1*, *Tktl1*, and *Ehhadh*, and that these genes exhibited an overall negative correlation trend with the above metabolites. Taken together, this cross-omics pattern may be consistent with alterations in glycolytic and alternative metabolic processes, while the upregulation of ETHE1 may reflect an oxidative-stress-associated response [27]. In biosynthesis of secondary metabolites, the increased abundance of phosphomannomutase 1 (PMM1) at the protein level was accompanied by broad reductions in multiple amino acids, including L-aspartic acid, L-lysine, and L-asparagine, as well as metabolites such as riboflavin and serotonin, were broadly downregulated. Moreover, *Tktl1* showed significant negative correlations with L-aspartic acid, L-lysine, and riboflavin, further connecting the transcriptomic and metabolomic responses within this pathway. These amino-acid-related molecular changes may reflect stress-associated metabolic adjustments related to the conservation of energy and carbon skeleton resources [28]. In addition, Qiu et al. reported that DTX1 exposure progressively reduced fatty acid content and altered the activities of lipid-metabolizing enzymes [29]. Together with the coordinated molecular changes observed across the transcriptomic, proteomic, and metabolomic levels, these findings suggest that DTX1 exposure is associated with extensive multi-layered metabolic alterations.

AZA3 exhibited intermediate cytotoxicity, and its molecular perturbations were mainly associated with lipid metabolism. Both primary bile acid biosynthesis and glycosphingolipid biosynthesis are closely associated with lipid metabolic processes. In primary bile acid biosynthesis, AZA3 treatment upregulated *Hsd3b7*, a bile-acid biosynthesis-related gene [30], and downregulated *Cyp46a1*, a cholesterol catabolism-related gene [31], suggesting potential alterations in cholesterol- and bile-acid-related metabolic processes. In glycosphingolipid biosynthesis, *A4galt* and *B3gnt4* were upregulated, whereas *B3gnt3* and *St3gal4* were downregulated, indicating alterations in genes associated with glycosphingolipid biosynthesis that may be related to membrane-associated recognition and signaling processes [32]. The coordinated gene-expression changes associated with these two lipid metabolism-related pathways are consistent with a previous toxin-related study by Bodero et al. showing that AZA1 upregulates genes involved in cholesterol biosynthesis [33], while AZA1-induced JNK activation and cell-volume decrease in primary cultured neurons further support AZA-related stress-response perturbations in a neuronal model [34].

YTX also exhibited intermediate cytotoxicity, with its molecular responses mainly associated with protein processing and endoplasmic reticulum homeostasis. Within the biosynthesis of nucleotide sugars pathway, transcriptomic and proteomic changes provided complementary cross-omics evidence. At the transcriptomic level, *Nagk*, *Galk2*, and *Uap1l1* were upregulated, whereas *Uap1* and *Nans* were downregulated; proteomic analysis further showed that HK2, PMM1, and UAP1L1 were upregulated. Correlation analysis revealed that *Nagk* was significantly positively correlated with HK2 and UAP1L1, *Uap1* was positively correlated with UAP1L1, whereas *Uap1l1* was negatively correlated with UAP1L1, suggesting that UAP1L1 may be differentially regulated by distinct pyrophosphorylase isoforms. A similar cross-omics pattern was observed in protein export, where changes in endoplasmic-reticulum-related genes occurred together with altered abundance of a mitochondrial protein and gene–protein correlation trends. *Arxes1* was upregulated, whereas the endoplasmic reticulum chaperone *Hspa5*, the translocation channel subunit *Sec62*, and the signal recognition particle component *Srp72* were downregulated. Meanwhile, OXA1L, an inner mitochondrial membrane protein, was upregulated and showed positive correlations with *Hspa5*, *Sec62*, and *Srp72*. The downregulation of *Hspa5*, which is involved in protein-folding-related processes [35], together with the decreases in *Sec62* and *Srp72*, suggests alterations in endoplasmic reticulum protein-folding and translocation-related processes [36,37]. The simultaneous molecular changes and cross-omics correlations therefore support coordinated responses involving endoplasmic reticulum and mitochondrial protein transport-related processes [38]. YTX has been shown to induce ER-stress-mediated autophagic cell death and apoptotic changes in BE(2)-M17 neuroblastoma cells, including mitochondrial membrane potential loss and caspase-3 activation, supporting the relevance of mitochondrial stress-related responses in neuronal models [26]. Together, these findings provide transcriptomic–proteomic support for YTX-associated alterations in nucleotide-sugar biosynthesis and endoplasmic reticulum–mitochondrial protein transport.

In contrast to the other three toxins, PTX2 exhibited the weakest cytotoxicity and the most limited molecular response. Despite this relatively restricted response, tyrosine metabolism emerged as the only pathway supported by differential molecules at both the transcriptomic and metabolomic levels. Specifically, *Dbh*, *Aox1*, *Aox4*, and *Ddc* were upregulated, whereas the metabolite 3,4-dihydroxy-L-phenylalanine (DOPA) was downregulated and showed a negative correlation with *Aox4*. *Ddc* is an enzyme involved in dopamine synthesis [39], whereas *Aox* participates in the oxidative metabolism of catecholamine-related compounds [40]. This inverse gene–metabolite pattern is consistent with altered dopamine- and catecholamine-related metabolism following PTX2 exposure. In addition, within the cGMP–PKG signaling pathway, PTX2 treatment was associated with changes in both positive and negative regulatory nodes. The cGMP synthase *Gucy1a2* and *Plcb4* were upregulated, whereas the myosin light chain gene *Myl9* and the serum response factor gene *Srf* were downregulated. The former changes were associated with cGMP- and calcium-related signaling, whereas the latter may indicate alterations in cellular contractility-related processes [41,42]. The simultaneous perturbation of both positive and negative regulatory nodes may reflect a compensatory cellular response to PTX2 exposure, although the underlying mechanism remains unclear. PTX2 is known mainly to disrupt cytoskeletal integrity through binding to actin [18]. Consistent with this cytoskeleton-centered mechanism, PTX6, a derivative of PTX2, has been shown to induce time- and dose-dependent F-actin depolymerization in neuroblastoma cells [43]. The present study further showed that PTX2 was also associated with alterations in tyrosine metabolism and cGMP–PKG signaling, suggesting that its cytotoxicity-associated molecular response pattern may be more complex than previously recognized.

**Figure 8:**
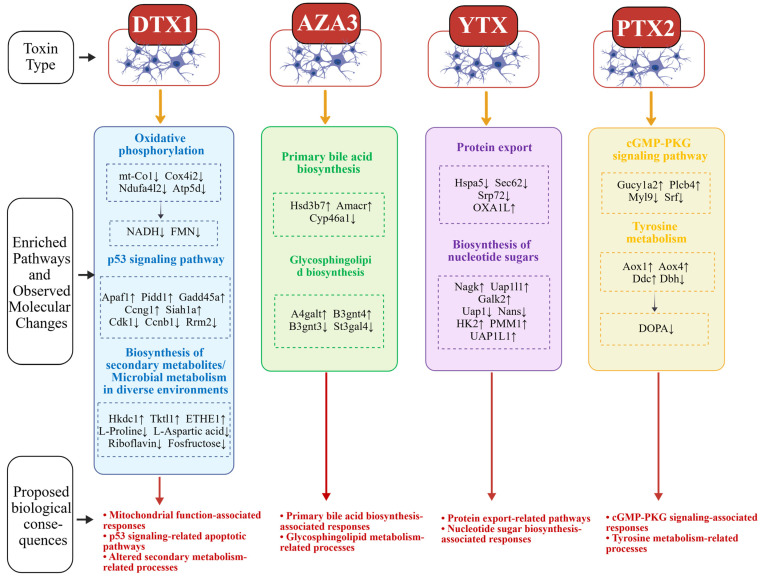
Schematic illustration of the distinct molecular perturbation patterns induced by four LMTs (DTX1, AZA3, YTX, and PTX2) in Neuro-2a cells. Arrows indicate upregulation (↑) or downregulation (↓) of the corresponding molecules.

Taken together, the four toxins differed in cytotoxic potency and also exhibited distinct molecular perturbation patterns in Neuro-2a cells. DTX1 was mainly associated with pathways related to mitochondrial energy metabolism and the p53-mediated stress pathway, with its effects further extending to carbohydrate, lipid, and amino acid metabolic networks. AZA3 was mainly associated with lipid metabolism-related molecular alterations. YTX was characterized primarily by alterations associated with endoplasmic reticulum protein processing and glycosylation-donor biosynthesis. In contrast, PTX2 mainly involved tyrosine metabolism and cGMP–PKG signaling network (Figure 8).

### 3.2. Shared Immune-Related and Cytoskeleton-Associated Responses Induced by AZA3 and YTX

DTX1, AZA3, and YTX all showed enrichment of DEGs in metabolism-related pathways, such as the pentose phosphate pathway, metabolic pathways, biosynthesis of amino acids, and the HIF-1 signaling pathway, suggesting that metabolism-related pathway enrichment may represent a shared molecular response associated with three toxin treatments. In addition to metabolism-related pathways, DEGs in the AZA3 and YTX groups were also commonly enriched in the IL-17 signaling pathway, regulation of actin cytoskeleton, and ECM-receptor interaction, indicating a high degree of similarity in their transcriptional regulatory patterns.

Within the IL-17 signaling pathway, both AZA3 and YTX induced upregulation of *Rela*, which encodes the p65 subunit of NF-κB. However, unlike the canonical activation pattern, *Fosb* expression was suppressed, which may be associated with alterations in AP-1-related transcriptional responses [44]. AP-1 plays a key regulatory role in cellular stress and immune responses [45]. Proteomic analysis further showed that TBK1 was significantly upregulated in the YTX group. Correlation analysis revealed that TBK1 was positively correlated with *Fosb* but negatively correlated with *Rela*, suggesting that TBK1 may be associated with AP-1-related responses in this pathway rather than with canonical NF-κB signaling. Reale et al. previously reported that both YTX and AZA1 could induce NF-κB nuclear translocation [46], and Franchini et al. also observed that YTX could trigger inflammatory responses [47]. In contrast to those studies, which mainly focused on NF-κB, the present study identified an association between TBK1 and AP-1-related molecules, suggesting that AZA3 and YTX may be associated with immune-related signaling involving AP-1.

Integrins serve as a bridge linking the extracellular matrix (ECM) to the actin cytoskeleton, thereby functionally coupling ECM–receptor interaction and regulation of actin cytoskeleton [48,49]. In the present study, both AZA3 and YTX broadly downregulated the expression of integrin subunits (*Itgb4* and *Itga1*), ECM components (*Fn1*, *Col4a1*, and *Col4a2*), and the focal adhesion protein *Vcl*, suggesting coordinated molecular alterations along the signaling axis from ECM receptors to intracellular cytoskeletal anchoring. Meanwhile, both toxins induced upregulation of *Pip5k1c* and *Fgd3*, which may represent a compensatory response. Proteomic data further showed that actin-related protein 2/3 complex subunit 3 (ARPC3) was upregulated in the AZA3 group, whereas IQGAP1, a key scaffold protein of the actin cytoskeleton, was upregulated in the YTX group. Correlation analysis showed that integrin- and cytoskeleton-related genes showed overall positive correlations with the above proteins, with selected correlations reaching statistical significance. This is consistent with the findings of Korsnes et al., who reported that YTX induces depolymerization of the F-actin cytoskeleton and cleavage of the focal adhesion protein tensin [50]. In addition, the present study further showed that AZA3 was also associated with alterations in cytoskeleton-related pathways. Notably, in the AZA3 group, ECM components showed discordant changes between the transcriptomic and proteomic datasets. At the transcriptomic level, collagen genes (*Col4a1* and *Col4a2*) and integrin subunit genes were broadly downregulated, whereas at the proteomic level, several collagens (COL1A, COL2A, and COL6A) and thrombospondins (THBS2, THBS3, and THBS4) were significantly upregulated. This transcript–protein discordance indicates that changes in transcript abundance did not directly translate into corresponding protein-level changes at the 48h sampling point. Such differences may reflect post-transcriptional regulation, differences in translation efficiency or protein turnover, and temporal delays between mRNA and protein responses [51]. Therefore, the transcriptomic and proteomic results were interpreted as complementary molecular responses rather than as a direct one-to-one relationship. The upregulation of thrombospondins may, in turn, represent a compensatory expression response associated with ECM-related alterations [52].

Although AZA3 and YTX showed partially similar molecular responses in Neuro-2a cells, they differ in their reported organism-level toxicological profiles. AZA3 belongs to the azaspiracid group, for which human intoxications are mainly characterized by gastrointestinal symptoms, and AZA1–AZA3 have also been shown to cause acute oral toxicity in mice [13,53]. In contrast, YTXs are not associated with a well-defined human diarrhetic syndrome and have been proposed to be excluded from the diarrhetic shellfish poisoning toxin group, while their toxicity in mice is strongly route dependent, being much lower after oral exposure than after intraperitoneal injection [54,55]. Thus, the shared alterations in IL-17 signaling, ECM–receptor interaction, and regulation of the actin cytoskeleton observed here may represent common downstream cytotoxic response patterns in Neuro-2a cells, rather than direct reflections of toxin-specific symptoms in humans or mice.

**Figure 9:**
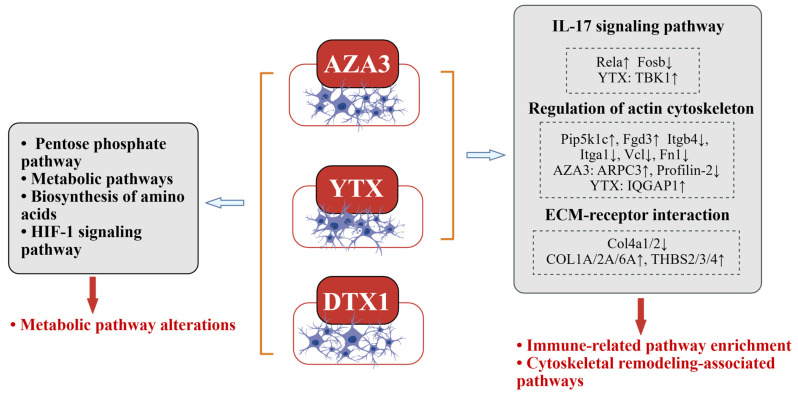
Conceptual model of common metabolic responses among DTX1, AZA3, and YTX and shared immune- and cytoskeleton-related responses between AZA3 and YTX in Neuro-2a cells. Arrows indicate upregulation (↑) or downregulation (↓) of the corresponding molecules.

Taken together, DTX1, AZA3, and YTX all exhibited metabolism-related molecular responses as a common background, whereas AZA3 and YTX showed additional overlap in immune-related and cytoskeletal-associated pathways (Figure 9). Overall, the pathway-specific correlation analyses in Tables S3–S6 support the cross-omics consistency of the molecular response patterns summarized in Figure 8 and Figure 9; however, these correlations provide associative evidence rather than independent functional validation of the candidate biological processes.

### 3.3. Correspondence Between Molecular Perturbation Patterns and Acute Cytotoxic Potency

The molecular perturbation patterns induced by the four LMTs in Neuro-2a cells generally corresponded to their acute cytotoxic potencies. As the most cytotoxic toxin, DTX1 was associated with a broader range of enriched pathways and molecular functional modules, including pathways associated with mitochondrial energy metabolism and the p53-centered stress response, as well as multi-layered metabolic networks such as carbohydrate and amino acid metabolism. AZA3 and YTX exhibited intermediate acute cytotoxicity, and their molecular responses likewise were associated with metabolism-related pathways, while also displaying distinct features of lipid metabolic remodeling and protein processing, respectively, together with shared immune-related and cytoskeleton-associated alterations. In contrast, PTX2, which showed the weakest cytotoxicity, exhibited relatively few enriched pathways and differential molecules, with its molecular responses being mainly associated with tyrosine metabolism and cGMP–PKG signaling network. These results indicate that the differences in acute cytotoxicity among LMTs are reflected not only in their cytotoxic potency, but also in their molecular perturbation patterns and associated functional modules.

It should be noted that the correspondence revealed in the present study, based on omics-derived candidate pathways and molecular associations, mainly reflects an overall trend rather than a strict one-to-one relationship. In other words, greater numbers of DEGs, enriched pathways, and associated functional modules do not necessarily translate into stronger toxicity, nor do they imply a direct causal relationship. Moreover, previous studies have shown that transcriptome–proteome relationships are often only partially concordant, and enrichment-based pathway results should therefore be interpreted as candidate biological processes rather than direct functional evidence [51,56]. Different toxins may differ substantially in their candidate molecular targets, signaling-related responses, and cellular compensatory capacity. In addition, the present study was conducted using three biological replicates per group, a single time point, and a single cell model, which may limit statistical power and is insufficient to fully capture the dynamic effects of these toxins at different stages of exposure or in other cellular models. Moreover, many cited mechanistic studies were conducted in experimental systems other than Neuro-2a cells and therefore provide supporting context rather than direct validation of the proposed pathways. Therefore, further functional validation using targeted pathway interventions, multicellular models, or in vivo experiments is required to clarify the biological roles of these candidate pathways in acute cytotoxicity.

## 4. Conclusions

This study compared the acute cytotoxicity and multi-omics molecular perturbation patterns induced by four representative LMTs in Neuro-2a cells. The results showed that DTX1, which exhibited the strongest cytotoxicity, was associated with the greatest number of enriched pathways and functional modules, including pathways related to mitochondrial energy metabolism, the p53-mediated stress response, and multi-layered metabolic networks. AZA3 and YTX, which showed intermediate cytotoxicity, were mainly associated with enriched pathways and molecular alterations related to cytoskeleton-associated, immune-related, and metabolism-related processes, and displayed partially similar perturbation patterns. In contrast, PTX2, which exhibited the weakest cytotoxicity, was mainly associated with tyrosine metabolism and the cGMP–PKG signaling network, and involved the fewest pathways and functional modules. Overall, the molecular perturbation patterns of different LMTs generally corresponded to their acute cytotoxic potencies, while each toxin exhibited distinct enriched pathways and associated functional modules. Among the four toxins, AZA3 and YTX showed the most similar molecular perturbation patterns. This study provides a multi-omics basis for comparing cytotoxicity-associated molecular responses induced by different classes of LMTs in Neuro-2a cells and lays the foundation for subsequent functional validation. Further targeted functional studies are required to validate the biological roles of the candidate pathways identified by the multi-omics analyses.

## 5. Materials and Methods

### 5.1. Chemicals and Solutions

Certified reference materials (CRMs) of DTX1 (7.8 ± 0.5 µg/mL), AZA3 (1.04 ± 0.04 µg/mL), YTX (4.9 ± 0.2 µg/mL), and PTX2 (4.40 ± 0.13 µg/mL) were purchased from the National Research Council of Canada, Institute for Marine Biosciences (NRC CNRC, Halifax, NS, Canada). All toxins are lipophilic and were dissolved in methanol in 1 mL ampoules, accompanied by certificates of quality control analysis. After the experiments, waste containing toxins or other hazardous substances was handled by a certified professional company for safe disposal.

### 5.2. Cell Culture and Toxin Exposure

The neuroblastoma cell line Neuro-2a (SCSP-5035) was obtained from the Stem Cell Bank, Chinese Academy of Sciences (Beijing, China). Neuro-2a cells were maintained in minimum essential medium (MEM) supplemented with 10% fetal bovine serum (FBS), 1% sodium pyruvate (100 mM), 1% GlutaMAX^TM^, 1% non-essential amino acids (NEAA, 100×), and 0.5% antibiotic solution (10 mg/mL streptomycin and 1000 U/mL penicillin). All media and supplements were purchased from Gibco (Grand Island, NY, USA). Toxin stock solutions were dissolved in culture medium supplemented with 10% FBS and serially diluted as required. Cells were passaged weekly using 0.25% trypsin-EDTA solution (0.25% trypsin, 0.02% EDTA) for detachment, and cultured at 37 °C with 5% CO_2_ in a Bluepard BPN-190CH (UV) incubator (Bluepard, Shanghai, China) until reaching near-confluence (~100%) between passages.

Neuro-2a cells were seeded in 96-well plates at an initial density of 20,000 cells per well. Before toxin exposure, all wells were examined under an inverted light microscope and confirmed to contain fully attached, near-confluent cells (~100% confluence) to minimize variability in cell status. Based on previous studies, DTX1, AZA3, YTX, and PTX2 were selected as representative toxic components for each group due to their pronounced cytotoxicity toward Neuro-2a cells [6]. After 72 h of incubation, the marine toxin standards were serially diluted in MEM (DTX1/YTX: 2, 4, 8, 16, 32, 64 nM; PTX2/AZA3: 1, 2, 4, 8, 16, 32 nM), covering the expected effective concentration range for each toxin, and 100 μL of the corresponding concentration was added to each well. To minimize matrix effects, 100 μL of methanol (diluted in 10% FBS medium to match the solvent proportion of the toxins) was added to designated control wells. All treatments, including solvent controls, were performed in triplicate biological replicates. Cell viability was measured at 24 h and 48 h post-exposure using the MTT assay. Throughout the experiment, cells were maintained under constant physiological conditions (37 °C, 5% CO_2_).

### 5.3. Cell Viability Assay

At the end of the 24 h and 48 h incubation periods, cell viability was quantified using the MTT colorimetric assay according to established protocols [19]. MTT reagent (Sigma-Aldrich, St. Louis, MO, USA) was prepared as a 5 mg/mL stock solution in phosphate-buffered saline (PBS) and subsequently diluted 1:6 with serum-free culture medium. After treatment, the culture medium was carefully removed and replaced with 60 μL of the MTT-serum-free medium mixture per well, yielding a final MTT concentration of 0.8 mg/mL.

Following 4 h of metabolic conversion at 37 °C in 5% CO_2_, the MTT-containing medium was removed, and the formazan crystals formed inside the cells were dissolved in 100 μL dimethyl sulfoxide (DMSO). The plate was then subjected to orbital shaking (600 rpm for 10 min) using a microplate shaker (Bluepard, Shanghai, China) to ensure complete dissolution of the crystals. Absorbance was measured at 455 nm using a Multiskan FC microplate reader (Thermo Fisher Scientific, Waltham, MA, USA). All experimental procedures were conducted under sterile and light-protected conditions.

### 5.4. Multi-Omics Sample Collection

Neuro-2a cells were exposed to 3 nM DTX1, 4 nM AZA3, 6 nM YTX, or 12 nM PTX2 for 48 h. These exposure concentrations were selected based primarily on the 48 h dose–response results and IC_50_ estimates obtained in the present study, as this cytotoxicity-based dose-selection strategy has also been used in previous studies of LMTs in Neuro-2a cells [6,19]. Specifically, the selected concentrations were close to the estimated 48 h IC_50_ values and within the corresponding 95% confidence intervals, thereby supporting comparative multi-omics analyses under near-equipotent cytotoxic exposure conditions rather than environmentally equivalent exposure concentrations. After exposure, cells from each toxin-treated group and the solvent control group were collected, with three biological replicates per group, for integrated multi-omics analysis. Following the manufacturer’s instructions, culture medium was removed at the end of the exposure period, and 2 mL of TRIzol^®^ Reagent (Invitrogen, Waltham, MA, USA) was added per 10 cm^2^ of culture area. The culture plates were gently rotated several times to ensure thorough contact between TRIzol and the cells. The resulting cell lysates were transferred to RNase-free tubes and repeatedly aspirated using a disposable syringe until no visible cell clumps remained, yielding a clear, non-viscous solution fully dissolved in TRIzol. Samples were then stored at −80 °C until further use.

### 5.5. Transcriptome Analysis

Total RNA was extracted using TRIzol^®^ Reagent according to the manufacturer’s instructions, and genomic DNA contamination was removed using DNase I (TaKaRa, Beijing, China). RNA concentration and purity were assessed with a NanoDrop 2000 spectrophotometer (Thermo Fisher Scientific, Waltham, MA, USA), and RNA integrity was evaluated using an Agilent 2100 Bioanalyzer (Agilent Technologies, Santa Clara, CA, USA). Samples meeting quality criteria (OD260/280 = 1.8–2.2, OD260/230 ≥ 2.0, RIN ≥ 6.5, 28S:18S ≥ 1.0, total RNA > 10 μg) [57] were used for library preparation. Between 1 and 4 μg of total RNA per sample was used as input for poly(A)+ mRNA enrichment with mRNA capture beads, followed by fragmentation (~250 bp), cDNA synthesis, adapter ligation, and PCR amplification to construct sequencing libraries. Libraries passing quality control on the Agilent 2100 Bioanalyzer were sequenced on the DNBSEQ-T7 platform (BGI, Shenzhen, China) using paired-end 150 bp reads.

Raw sequencing data were quality-controlled and filtered to remove adapter sequences, low-quality reads, reads with high N content, and reads that were too short, generating clean reads. Clean reads were aligned to the mouse reference genome (GRCm39) using HISAT2, and mapping quality was assessed with Qualimap 2.2.1. Gene-level read counts were obtained using featureCounts 2.0.6, and transcript abundance was calculated as Fragments Per Kilobase of transcript per Million mapped reads (FPKM). FPKM values were used as transcript abundance metrics for expression-level visualization, PCA, and low-expression gene filtering because they normalize read counts by both transcript length and sequencing depth [58]. PCA was performed on the normalized expression matrix (FPKM) using R 4.2.3 to evaluate the overall transcriptomic differences among treatment groups and the control, as well as the consistency of biological replicates. Low-expression genes were filtered out, retaining only those with max (FPKM) ≥ 1 in at least one sample. Differential expression analysis was performed using edgeR, with genes satisfying |log_2_ fold change (log_2_FC)| ≥ 1 and false discovery rate (FDR) ≤ 0.05 defined as DEGs. Hierarchical clustering was performed on DEGs to explore expression patterns, followed by Gene Ontology (GO) functional enrichment and KEGG pathway enrichment analyses using Goatools 1.4.6 and KOBAS 3.0, respectively. *p* < 0.05 after multiple testing correction were considered statistically significant.

The raw transcriptomic sequencing data have been deposited in the NCBI Sequence Read Archive (SRA) under BioProject accession number PRJNA1460527.

### 5.6. Label-Free Quantitative Proteomic Analysis

Proteins were extracted from three biological replicates per group. Samples were lysed with DB protein lysis buffer (6 M urea, 100 mM TEAB, pH 8.5) and centrifuged to collect the supernatant. Proteins were reduced with dithiothreitol and alkylated with iodoacetamide. Protein concentration was measured using the Bradford Protein Assay Kit (Beyotime, Shanghai, China).

Equal amounts of protein from each sample were digested with trypsin in two steps. After digestion, the reaction was terminated with formic acid, and the supernatant was collected by centrifugation. Peptides were desalted using C18 columns, eluted, and lyophilized for subsequent analysis.

Liquid chromatography-tandem mass spectrometry (LC-MS/MS) was performed using a Vanquish^TM^ Neo Ultra-High Performance Liquid Chromatography system coupled with an Orbitrap Astral mass spectrometer (Thermo Fisher Scientific, Waltham, MA, USA), employing a Data-Independent Acquisition (DIA) mode. Raw mass spectrometry data were processed using DIA-NN for database search and quantification, with the Mus_musculus_uniprot_2025_06_11_Swissprot.fasta (17,237 sequences) database. FDR validation was applied to ensure reliable protein identification. iRT peptides were spiked into samples for retention time calibration, generating a protein quantification matrix. DEPs between groups were identified using a *t*-test, with thresholds of FDR ≤ 0.05 and |log_2_FC| ≥ 1, corresponding to FC ≥ 2 or FC ≤ 0.5. DEPs were used for subsequent bioinformatic analyses. KEGG pathway enrichment was considered significant at *p* < 0.05.

The mass spectrometry proteomics data have been deposited to the ProteomeXchange Consortium [https://proteomecentral.proteomexchange.org (accessed on 11 May 2026)] via the iProX partner repository [59,60] with the dataset identifier PXD078169.

### 5.7. Untargeted LC-MS Metabolomic Analysis

Samples were placed in Eppendorf tubes (Eppendorf SE, Hamburg, Germany) and resuspended in pre-cooled 80% methanol containing 0.1% formic acid, followed by ultrasonic extraction. After centrifugation, the supernatants were lyophilized and reconstituted in 10% methanol. The resulting solution was used for LC-MS/MS analysis. Quality control (QC) samples were prepared by pooling equal volumes from all experimental samples. Blank samples were prepared using 53% methanol in water in place of the experimental samples and processed in the same manner to remove background ions.

Chromatographic separation was performed using a Hypersil Gold C18 column, with mobile phase A consisting of 0.1% formic acid in water and mobile phase B consisting of methanol. Mass spectrometry data were acquired in both positive and negative electrospray ionization modes, and MS/MS fragmentation spectra were obtained using data-dependent acquisition.

Raw data were imported into Compound Discoverer 3.3 software (Thermo Fisher Scientific, Waltham, MA, USA) for peak detection, alignment, and relative quantification. Peak areas were normalized using the first QC sample. Molecular formulas were predicted based on molecular and fragment ions and matched against the mzCloud, mzVault, and Masslist databases for metabolite identification, with background ions removed using blank samples. Features with a coefficient of variation > 30% in QC samples were excluded to obtain the final metabolite identification and relative quantification dataset.

Metabolites were annotated based on KEGG, HMDB, and LIPIDMaps databases. Partial least squares discriminant analysis was performed using metaX 1.4.2 to calculate variable importance in projection (VIP) scores. Univariate analysis was performed using *t*-tests to obtain *p* values and calculate FC between groups. DEMs were defined using thresholds of VIP ≥ 1, *p* ≤ 0.05, and |log_2_FC| ≥ 1, corresponding to FC ≥ 2 or FC ≤ 0.5. Metabolic pathway enrichment was assessed based on KEGG, with pathways considered significantly enriched at *p* < 0.05.

The raw metabolomic data have been deposited in OMIX, China National Center for Bioinformation/Beijing Institute of Genomics, Chinese Academy of Sciences, under accession number OMIX016675.

### 5.8. Integrated Multi-Omics Analysis

To systematically characterize the shared and specific cytotoxicity-associated molecular responses induced by four LMTs (DTX1, AZA3, YTX, and PTX2) in Neuro-2a cells, pathways were prioritized for biological interpretation when they were significantly enriched in the transcriptomic analysis and further supported by at least one of the following features: corresponding DEPs or DEMs mapped to the same pathway, relevance to the observed cytotoxicity ranking, or representation of toxin-specific or shared response patterns. For these prioritized pathways, Spearman correlation analyses were performed between DEGs and DEPs, as well as between DEGs and DEMs, to evaluate the association strength across omics layers. Raw *p*-values obtained from the correlation analyses were adjusted within each pathway-specific correlation matrix using the Benjamini–Hochberg false discovery rate (FDR) method, and correlations with FDR-adjusted *p* < 0.05 were considered statistically significant.

### 5.9. RT-qPCR Validation

To validate the transcriptome data, eight representative DEGs associated with toxin-specific enriched pathways were selected for RT-qPCR analysis: *Cox4i2* and *Gadd45a* for the DTX1 group, *Hsd3b7* and *A4galt* for the AZA3 group, *Hspa5* and *Nagk* for the YTX group, and *Myl9* and *Gucy1a2* for the PTX2 group. Gene expression levels were normalized to the housekeeping gene *β-actin* [61]. Specific primers for β-actin and the eight target genes were designed via NCBI and synthesized by Sangon Biotech (Shanghai, China) Co., Ltd. (Table S1). Quantitative PCR was performed using the QuantStudio 7 Pro Real-Time PCR System (Thermo Fisher Scientific, Waltham, MA, USA), and relative gene expression levels were calculated using the 2^−ΔΔCt^ method.

### 5.10. Data Analysis

Statistical analyses were performed using SPSS 19 (IBM Corp., Armonk, NY, USA). All data are presented as mean ± SD. IC_50_ values were calculated using probit analysis. Comparisons between experimental and control groups were performed using one-way ANOVA for normally distributed data, followed by Tukey’s HSD test for multiple comparisons. Figures were generated using GraphPad Prism 10.1.2 (GraphPad Software, New York, NY, USA) and Origin 2021 (OriginLab, Northampton, MA, USA). The graphical abstract was created with BioGDP.com (accessed on 12 June 2026) [62]. Statistical significance was set at *p* < 0.05.

## Figures and Tables

**Table 1:** Half-maximal inhibitory concentration (IC_50_) values of Neuro-2a cells exposed to four lipophilic marine toxins (LMTs) for 24 and 48 h.

Exposure Time	DTX1	AZA3	YTX	PTX2
24 h	3.39 ± 1.00 ^d^ (1.46–7.37)	9.67 ± 0.34 ^c^ (8.86–10.55)	21.88 ± 4.72 ^b^ (12.14–38.12)	26.77 ± 1.76 ^a^ (22.71–31.48)
48 h	2.62 ± 0.88 ^c^ (1.14–5.63)	4.32 ± 0.65 ^bc^ (3.00–6.15)	7.45 ± 0.71 ^ab^ (5.84–9.44)	10.55 ± 4.65 ^a^ (3.10–31.20)

IC_50_ values were calculated using probit analysis. Dinophysistoxin-1 (DTX1), azaspiracid-3 (AZA3), yessotoxin (YTX), and pectenotoxin-2 (PTX2) were tested. Different lowercase letters within the same row indicate significant differences among toxin treatment groups according to Tukey’s honestly significant difference (HSD) test (*p* < 0.05). Data are presented as mean ± standard deviation (SD) (*n* = 3), with 95% confidence intervals shown in parentheses. The 95% confidence intervals were calculated from log-transformed IC_50_ values and back-transformed to the original concentration scale.

## Data Availability

The raw transcriptomic, proteomic, and metabolomic data generated in this study have been deposited in public repositories. The transcriptomic data have been deposited in the NCBI Sequence Read Archive (SRA) under BioProject accession number PRJNA1460527. The mass spectrometry proteomics data have been deposited in the ProteomeXchange Consortium via the iProX partner repository with the dataset identifier PXD078169. The metabolomic data have been deposited in OMIX, China National Center for Bioinformation/Beijing Institute of Genomics, Chinese Academy of Sciences, under accession number OMIX016675.

## Supplementary Materials

The following supporting information can be downloaded at: https://www.mdpi.com/article/10.3390/toxins18070274/s1, Figure S1. Fitted dose–response curves of DTX1, AZA3, YTX, and PTX2 in Neuro-2a cells; Figure S2. KEGG Pathway Enrichment Analysis of Differentially Expressed Proteins (DEPs) in Neuro-2a Cells Exposed to DTX1, AZA3, and YTX; Figure S3. KEGG pathway enrichment analysis of differentially expressed metabolites (DEMs) in Neuro-2a cells exposed to DTX1, AZA3, YTX, and PTX2; Figure S4. Complete Spearman correlation heatmap of all DEGs with DEPs or DEMs in biosynthesis of secondary metabolites in DTX1-treated Neuro-2a cells; Figure S5. Complete Spearman correlation heatmap of all DEGs with DEPs or DEMs in microbial metabolism in diverse environments in DTX1-treated Neuro-2a cells; Figure S6. Spearman correlation scatter plot between all DEGs and DEM in Tyrosine metabolism (PTX2-treated Neuro-2a cells); Table S1. Primers used for RT-qPCR; Table S2. Summary of DEGs, DEPs and DEMs in key enriched pathways; Table S3. Spearman correlation coefficients between DEGs and DEMs in Oxidative phosphorylation (DTX1-treated Neuro-2a cells); Table S4. Spearman correlation coefficients between DEGs and DEPs in Regulation of actin cytoskeleton (AZA3-treated Neuro-2a cells); Table S5. Spearman correlation coefficients between DEGs and DEPs in ECM-receptor interaction (AZA3-treated Neuro-2a cells); Table S6. Spearman correlation coefficients between DEGs and DEPs in key enriched pathways in YTX-treated Neuro-2a cells: Regulation of actin cytoskeleton, IL-17 signaling pathway and Biosynthesis of nucleotide sugars and Protein export.

## Abbreviations

ARPC3	Actin-related protein 2/3 complex subunit 3
AZA3	Azaspiracid-3
DEGs	Differentially expressed genes
DEMs	Differentially expressed metabolites
DEPs	Differentially expressed proteins
DIA	Data-independent acquisition
DOPA	3,4-Dihydroxy-L-phenylalanine
DTX1	Dinophysistoxin-1
ECM	Extracellular matrix
ETHE1	Persulfide dioxygenase ETHE1, mitochondrial
FDR	False discovery rate
FMN	Riboflavin-5′-monophosphate
FPKM	Fragments per kilobase of transcript per million mapped reads
GO	Gene Ontology
IC_50_	Half-maximal inhibitory concentration
KEGG	Kyoto Encyclopedia of Genes and Genomes
LC-MS/MS	Liquid chromatography–tandem mass spectrometry
LMTs	Lipophilic marine toxins
log_2_FC	log_2_ fold change
MTT	3-(4,5-dimethylthiazol-2-yl)-2,5-diphenyltetrazolium bromide
NADH	Reduced nicotinamide adenine dinucleotide
PCA	Principal component analysis
PLS-DA	Partial least squares discriminant analysis
PMM1	Phosphomannomutase 1
PTX2	Pectenotoxin-2
QC	Quality control
RT-qPCR	Reverse transcription quantitative polymerase chain reaction
SD	Standard deviation
SRA	Sequence Read Archive
TBK1	TANK-binding kinase 1
VIP	Variable importance in projection
YTX	Yessotoxin
