# Supplementary material for: Integrated Multi-Omics Analysis Reveals Complex Cytotoxicity-Associated Molecular Response Patterns of Representative Toxins from Four Classes of Lipophilic Algal Toxins in Neuro-2a Cells
Source: Toxins (Basel). 2026 Jun 23;18(7):274. doi: 10.3390/toxins18070274 (PMC13416731; doi:10.3390/toxins18070274)
Supplement: Supplementary file 1 [file toxins-18-00274-s001.zip › toxins-4342467-supplementary.pdf]

# Supplementary Materials: Integrated Multi-Omics Analysis Reveals Complex Cytotoxicity-Associated Molecular Response Patterns of Representative Toxins from Four Classes of Lipophilic Algal Toxins in Neuro-2a Cells

**Xueru Wei, Pengrui Ren, Junkai Feng, Jingyuan Shi, Peipei Zhang and Hongjun Li**

**Table S1.** Primers used for RT-qPCR.

| Gene symbol | Accession number | Primer sequence (5′-3′) | Product length (bp) |
|---|---|---|---|
| Cox4i2 | NM_053091 | CTGCCCGGAGTCTGGTAATG<br>CAGTCAACGTAGGGGGTCATC | 109 |
| Gadd45a | NM_007836 | CCGAAAGGATGGACACGGTG<br>TTATCGGGGTCTACGTTGAGC | 121 |
| Hsd3b7 | NM_001040684 | GGGAGCTGCGTGTCTTTGA<br>GTGGATGGTCTTTGGACTGGC | 197 |
| A4galt | NM_001004150 | TCTTCTTCCTAGAGACATCGGAC<br>CCCTTTCATCAGCACAACCA | 113 |
| Hspa5 | NM_001163434 | GACTGCTGAGGCGTATTTGG<br>AGCATCTTTGGTTGCTTGTCG | 97 |
| Nagk | NM_019542 | CACGGTCCAAAGTCCTTTTACT<br>GTCTGTGCCAATCAGCCAGT | 92 |
| Myl9 | NM_172118 | AGAGGGCTACGTCCAATGTCT<br>CTCCAGATACTCGTCTGTGGG | 167 |
| Gucy1a2 | NM_001033322 | CCTTGCCTCAGACGATACACA<br>GCAGTCCGAGTACATTAGCAGT | 195 |
| β-actin | NM_007393 | GTACCACCATGTACCCAGGC<br>AACGCAGCTCAGTAACAGTCC | 247 |

**Table S2.** Summary of DEGs, DEPs and DEMs in key enriched pathways.

| Toxin | Pathways | Type | Up | Down |
|---|---|---|---|---|
| DTX1 | Oxidative phosphorylation | DEG | *Lhpp* | *mt-Co1*、*Atp5d*、*Cox4i2*、*Ndufs2*、*Ndufa2*、*Atp5e*、*Cox7c*、*Ndufa6*、*Ndufb4*、*Atp5o*、*Ndufv3*、*Cox7a2l*、*Uqcrc1*、*Atp5c1*、*Ndufb11*、*Cox4i1*、*Ndufa3*、*Cox8a*、*Ndufa13*、*Atp6v1g1*、*Ndufa4l2*、*Cox6a1*、*Ndufa7*、*Atp5g2*、*Uqcrh* |
| | | DEM | | Riboflavin-5'-monophosphate、NADH |

| | | | |
|---|---|---|---|
| p53 signaling pathway | DEG | *Sfn*、*Apaf1*、*Ccng1*、*Pidd1*、*Ccne2*、*Atr*、*Gadd45a*、*Siah1a* | *Cdk1*、*Rrm2*、*Gtse1*、*Ccng2*、*Ccnb2*、*Ccnb1*、*Cdkn2a* |
| Biosynthesis of secondary metabolites | DEG | *Pnp*、*Hmox1*、*Arg1*、*Acot2*、*Ehhadh*、*Rfk*、*Prps1l3*、*Amy1*、*Ak3*、*Papss2*、*Nme7*、*Impa1*、*Nme6*、*Hkdc1*、*Tktl1*、*Acy1*、*Hdc*、*Glyctk*、*Il4i1*、*Dgkh*、*Nt5c2*、*Pdss1*、*Lpin3*、*Ddc*、*Eno4*、*Pcca*、*Gpd2*、*Pla2g2d*、*Nat2*、*Hao1*、*Ak5* | *Th*、*Elovl1*、*Lpin1*、*Tpi1*、*Ldha*、*Bckdha*、*Lss*、*Elovl5*、*Nsdhl*、*Cyp51*、*Mvd*、*Fdft1*、*Agpat4*、*Pgk1*、*Fdps*、*Pfkm*、*Ntpcr*、*dh2*、*Eno2*、*Pgam1*、*Hmgcr*、*Acat2*、*Dgkz*、*Gapdh*、*Amt*、*Pgls*、*Pla2g1b*、*Hmgcs1*、、*Aldoa*、*Cbs*、*Blvrb*、*Gadl1*、*Idh3b*、*Tecr*、*Azin2*、*Ass1*、*Gyg*、*Tkt*、*Entpd3*、*Gpi1*、*Phgdh*、*Acss2*、*Msmo1*、*Tm7sf2*、*Hsd17b10*、*Sqle*、*Pmvk* |
| | DEP | Phosphomannomutase 1 | / |
| | DEM | Ferulic acid | 2-Heptyl-4(1H)-quinolone、L-Lysine、L-Aspartic acid、Riboflavin-5'-monophosphate、L-Proline、L-Asparagine、Riboflavin、Fosfructose、3,4-Dihydroxy-L-phenylalanine、Indole、Serotonin、Vitamin B5、N-ACETYL-5-HYDROXYTRYPTAMINE、Phenyllactic acid、alpha-Linolenic acid、N-Desmethyl Galanthamine、Corydaline、Dihydrozeatin-O-glucoside、Brevianamide F |
| Microbial metabolism in diverse environments | DEG | *Hmgcll1*、*Hkdc1*、*Papss2*、*Uox*、*Aox1*、*Ehhadh*、*Glyctk*、*Hao1*、*Prps1l3*、*Acyp2*、*Nat2*、*Eno4*、*Pcca*、*Aox4*、*Tktl1* | *Aldoa*、*Pgam1*、*Tkt*、*Tpi1*、*Idh3b*、*Ldha*、*Gapdh*、*Eno2*、*Aldoc*、*Mpst*、*Acat2*、*Acss2*、*Pgk1*、*Phgdh*、*Gpi1*、*Idh2*、*Tkfc*、*Pfkm*、*Pgls* |
| | DEP | Persulfide dioxygenase ETHE1, mitochondrial | / |
| | DEM | 5-Aminovaleric acid、4-Pyridoxic acid、m-Coumaric acid、4-Formylsalicylic acid | 4-Methyl-3-oxoadipate、L-Lysine、L-Aspartic acid、Pyridoxal、Fosfructose、Pyridoxamine、N-Methyl-L- |

| | | | | |
|---|---|---|---|---|
| | | | | glutamate、P-Toluenesulfonic acid |
| AZA3 | IL-17 signaling pathway | DEG | *Defb4*、*Rela*、*Lcn2*、*Fadd* | *Fosb*、*Mmp9*、*Hsp90b1*、*Cxcl10* |
| | Regulation of actin cytoskeleton | DEG | *Pip5k1c*、*Fgd3* | *C7*、*Egfr*、*Itgb3*、*Itgb4*、*Vcl*、*Fn1*、*Fgf1*、*Rras*、*Bdkrb1*、*Itga1*、*F2r*、*Fgfr3*、*Myl9* |
| | | DEP | Actin-related protein 2/3 complex subunit 3、Kininogen-1、Prothrombin | Profilin-2 |
| | ECM-receptor interaction | DEG | / | *Cd47*、*Hmmr*、*Itgb3*、*Itgb4*、*Fn1*、*Hspg2*、*Col4a1*、*Col4a2*、*Npnt*、*Itga1*、*Lamb2* |
| | | DEP | Thrombospondin-4、Collagen alpha-1(I) chain、Collagen alpha-1(II) chain、Collagen alpha-2(VI) chain、Thrombospondin-2、Collagen alpha-1(VI) chain、Thrombospondin-3、Cartilage oligomeric matrix protein | / |
| | Primary bile acid biosynthesis | DEG | *Hsd3b7*、*Amacr* | *Cyp46a1* |
| | Glycosphingolipid biosynthesis - lacto and neolacto series | DEG | *A4galt*、*B3gnt4* | *B3gnt3*、*St3gal4* |
| YTX | IL-17 signaling pathway | DEG | *Defb4*、*Rela*、*Lcn2*、*Fadd* | *Fosb*、*Mmp9*、*Hsp90b1*、*Fos*、*Usp25* |
| | | DEP | Serine/threonine-protein kinase TBK1 | / |
| | Regulation of actin cytoskeleton | DEG | *Pip5k1c*、*Fgd3* | *C7*、*Egfr*、*Itgb4*、*Vcl*、*Fn1*、*Fgf1*、*Rras*、*Bdkrb1*、*Ssh1*、*Itga1*、*F2r*、*Fgfr3* |
| | | DEP | Ras GTPase-activating-like protein IQGAP1、Proteinase-activated receptor 1 | / |

| | | | | |
|---|---|---|---|---|
| | ECM-receptor interaction | DEG | / | *Cd47*、*Lama4*、*Hmmr*、*Itgb4*、*Fn1*、*Hspg2*、*Col4a1*、*Col4a2*、*Npnt*、*Itga1* |
| | Protein export | DEG | *Arxes1* | *Hspa5*、*Sec62*、*Srp72* |
| | | DEP | Mitochondrial inner membrane protein OXA1L | / |
| | Biosynthesis of nucleotide sugars | DEG | *Nagk*、*Uap1l1*、*Galk2* | *Uap1*、*Nans* |
| | | DEP | UDP-N-acetylhexosamine pyrophosphorylase-like protein 1、Hexokinase-2、Phosphomannomutase 1 | / |
| PTX2 | cGMP-PKG signaling pathway | DEG | *Plcb4*、*Gucy1a2*、*Oprd1*、*Irs1*、*Adra2b* | *Myl9*、*Srf*、*Nppb* |
| | Tyrosine metabolism | DEG | *Aox1*、*Aox4*、*Ddc* | *Dbh* |
| | | DEM | / | 3,4-Dihydroxy-L-phenylalanine |

**Table S3.** Spearman correlation coefficients between DEGs and DEMs in Oxidative phosphorylation (DTX1-treated Neuro-2a cells).

| Metabolite | *mt-Co1* | *Atp5d* | *Cox4i2* | *Ndufs2* | *Ndufa2* | *Atp5e* | *Ndufa7* |
|---|---|---|---|---|---|---|---|
| FMN | 0.657 | 0.714 | 0.886 | 0.771 | 0.771 | 0.714 | 0.771 |
| NADH | 0.657 | 0.886 | 0.714 | 0.943 | 0.943 | 0.886 | 0.771 |
| | *Atp5c1* | *Lhpp* | *Ndufb11* | *Cox4i1* | *Ndufa3* | *Cox8a* | *Atp5g2* |
| FMN | 0.771 | -0.943 | 0.714 | 0.714 | 0.714 | 0.771 | 0.771 |
| NADH | 0.771 | -0.771 | 0.886 | 0.886 | 0.886 | 0.943 | 0.943 |
| | *Ndufa6* | *Ndufb4* | *Atp5o* | *Ndufv3* | *Cox7a2l* | *Uqcrc1* | *Uqcrh* |
| FMN | 0.714 | 0.714 | 0.714 | 0.886 | 0.771 | 0.714 | 0.657 |
| NADH | 0.886 | 0.886 | 0.886 | 0.714 | 0.771 | 0.886 | 0.657 |
| | *Cox7c* | *Ndufa4l2* | *Cox6a1* | *Ndufa13* | *Atp6v1g1* | / | / |
| FMN | 0.714 | 0.886 | 0.714 | 0.771 | 0.657 | / | / |
| NADH | 0.886 | 0.714 | 0.886 | 0.771 | 0.657 | / | / |

FMN, Riboflavin-5'-monophosphate.

**Table S4.** Spearman correlation coefficients between DEGs and DEPs in Regulation of actin cytoskeleton (AZA3-treated Neuro-2a cells).

| Gene | ARPC3 | KNG1 | F2 | KNG1 |
|---|---|---|---|---|
| *C7* | 0.714 | 0.943* | 0.943* | -0.714 |
| *Egfr* | 0.771 | 0.829 | 0.886 | -0.771 |
| *Itgb3* | 1.000 | 0.771 | 0.771 | -1.000 |
| *Itgb4* | 0.714 | 0.600 | 0.600 | -0.714 |
| *Vcl* | 0.771 | 0.886 | 1.000 | -0.771 |
| *Fn1* | 0.943* | 0.714 | 0.829 | -.943* |
| *Pip5k1c* | -0.714 | -0.771 | -.943* | 0.714 |
| *Fgf1* | 0.714 | 0.943* | 0.943* | -0.714 |
| *Fgd3* | -0.771 | -0.714 | -0.657 | 0.771 |
| *Rras* | 1.000 | 0.771 | 0.771 | -1.000 |
| *Bdkrb1* | 0.771 | 0.829 | 0.886 | -0.771 |
| *Itga1* | 0.771 | 0.886 | 1.000 | -0.771 |
| *F2r* | 0.771 | 0.886 | 1.000 | -0.771 |
| *Fgfr3* | 0.829 | 0.771 | 0.600 | -0.829 |
| *Myl9* | 1.000 | 0.771 | 0.771 | -1.000 |

ARPC3, Actin-related protein 2/3 complex subunit 3; KNG1, Kininogen-1; F2, Prothrombin; KNG1, Profilin-2 (* *FDR-adjusted p* < 0.05).

**Table S5.** Spearman correlation coefficients between DEGs and DEPs in ECM-receptor interaction (AZA3-treated Neuro-2a cells).

| Gene | THBS4 | COL1A | COL2A | COL6A | THBS2 | COL6A | THBS3 | COMP |
|---|---|---|---|---|---|---|---|---|
| *Cd47* | 0.886 | 0.943* | 0.886 | 0.886 | 0.943* | 0.943* | 0.543 | 0.886 |
| *Hmmr* | 0.943* | 1.000 | 0.943* | 0.943* | 0.829 | 1.000 | 0.600 | 0.943* |
| *Itgb3* | 0.829 | 0.771 | 0.829 | 0.829 | 0.600 | 0.771 | 0.829 | 0.829 |

| | | | | | | | | |
|---|---|---|---|---|---|---|---|---|
| *Itgb4* | 0.714 | 0.600 | 0.714 | 0.714 | 0.771 | 0.600 | 0.886 | 0.714 |
| *Fn1* | 0.943* | 0.829 | 0.943* | 0.943* | 0.657 | 0.829 | 0.771 | 0.943* |
| *Hspg2* | 0.886 | 0.771 | 0.886 | 0.886 | 0.600 | 0.771 | 0.886 | 0.886 |
| *Col4a1* | 0.829 | 0.771 | 0.829 | 0.829 | 0.600 | 0.771 | 0.829 | 0.829 |
| *Col4a2* | 0.771 | 0.714 | 0.771 | 0.771 | 0.543 | 0.714 | 0.943* | 0.771 |
| *Npnt* | 0.714 | 0.771 | 0.714 | 0.714 | 0.771 | 0.771 | 0.714 | 0.714 |
| *Itga1* | 0.943* | 1.000 | 0.943* | 0.943* | 0.829 | 1.000 | 0.600 | 0.943* |
| *Lamb2* | 0.886 | 0.771 | 0.886 | 0.886 | 0.600 | 0.771 | 0.886 | 0.886 |

THBS4, Thrombospondin-4; COL1A, Collagen alpha-1(I) chain; COL2A, Collagen alpha-1(II) chain; COL6A, Collagen alpha-2(VI) chain; THBS2, Thrombospondin-2; COL6A, Collagen alpha-1(VI) chain; THBS3, Thrombospondin-3; COMP, Cartilage oligomeric matrix protein (* *FDR-adjusted p* < 0.05).

**Table S6.** Spearman correlation coefficients between DEGs and DEPs in key enriched pathways in YTX-treated Neuro-2a cells: Regulation of actin cytoskeleton, IL-17 signaling pathway and Biosynthesis of nucleotide sugars and Protein export.

| Pathway | Gene | IQGAP1 | PAR1 | TBK1 | UAP1L1 | HK2 | PMM1 | OXA1L |
|---|---|---|---|---|---|---|---|---|
| Regulation of actin cytoskeleton | *C7* | 0.886* | 0.829 | / | / | / | / | / |
| | *Egfr* | 0.943* | 0.943* | / | / | / | / | / |
| | *Itgb4* | 0.886* | 0.829 | / | / | / | / | / |
| | *Vcl* | 0.886* | 0.829 | / | / | / | / | / |
| | *Fn1* | 0.943* | 0.943* | / | / | / | / | / |
| | *Pip5k1c* | -0.714 | -0.657 | / | / | / | / | / |
| | *Fgf1* | 0.943* | 0.771 | / | / | / | / | / |
| | *Fgd3* | -0.600 | -0.600 | / | / | / | / | / |
| | *Rras* | 0.657 | 0.714 | / | / | / | / | / |
| | *Bdkrb1* | 0.829 | 0.714 | / | / | / | / | / |
| | *Ssh1* | 0.943* | 0.943* | / | / | / | / | / |
| | *Itga1* | 0.886* | 0.829 | / | / | / | / | / |
| | *F2r* | 0.886* | 0.829 | / | / | / | / | / |
| | *Fgfr3* | 0.600 | 0.771 | / | / | / | / | / |
| IL-17 signaling pathway | *Defb4* | / | / | -0.771 | / | / | / | / |
| | *Fosb* | / | / | 1.000* | / | / | / | / |
| | *Mmp9* | / | / | 0.600 | / | / | / | / |
| | *Hsp90b1* | / | / | 0.714 | / | / | / | / |
| | *Fos* | / | / | 0.657 | / | / | / | / |
| | *Usp25* | / | / | 0.714 | | / | / | / |
| | *Rela* | / | / | -0.600 | / | / | / | / |
| | *Lcn2* | / | / | -0.771 | / | / | / | / |
| | *Fadd* | / | / | -0.886 | / | / | / | / |
| Biosynthesis of nucleotide sugars | *Nagk* | / | / | / | 0.829 | 0.943* | 0.771 | / |
| | *Uap1* | / | / | / | 0.943* | 0.829 | 0.829 | / |
| | *Uap1l1* | / | / | / | -0.829 | -0.943* | -0.771 | / |
| | *Galk2* | / | / | / | -0.771 | -1.000* | -0.657 | / |

| | | | | | | | | |
|---|---|---|---|---|---|---|---|---|
| | *Nans* | / | / | / | 0.714 | 0.600 | 0.771 | / |
| Protein export | *Arxes1* | / | / | / | / | / | / | -0.714 |
| | *Hspa5* | / | / | / | / | / | / | 0.771 |
| | *Sec62* | / | / | / | / | / | / | 0.771 |
| | *Srp72* | / | / | / | / | / | / | 0.771 |

IQGAP1, Ras GTPase-activating-like protein IQGAP1; PAR1, Proteinase-activated receptor 1; TBK1, Serine/threonine-protein kinase TBK1; UAP1L1, UDP-N-acetylhexosamine pyrophosphorylase-like protein 1; HK2, Hexokinase-2; PMM1, Phosphomannomutase 1; OXA1L, Mitochondrial inner membrane protein OXA1L (* *FDR-adjusted* $p < 0.05$).

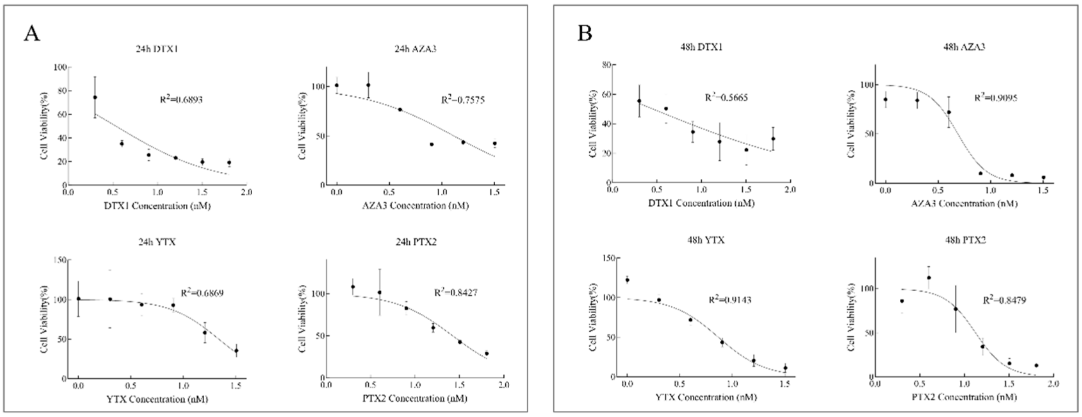


**Figure S1.** Fitted dose–response curves of DTX1, AZA3, YTX, and PTX2 in Neuro-2a cells. (A) Cells were exposed to the indicated toxins for 24 h. (B) Cells were exposed to the indicated toxins for 48 h. Symbols represent observed cell viability values, error bars indicate SD, and solid lines represent fitted dose-response curves. The x-axis is shown on a logarithmic concentration scale. $R^2$ values indicate the goodness of fit of the corresponding dose-response curves.

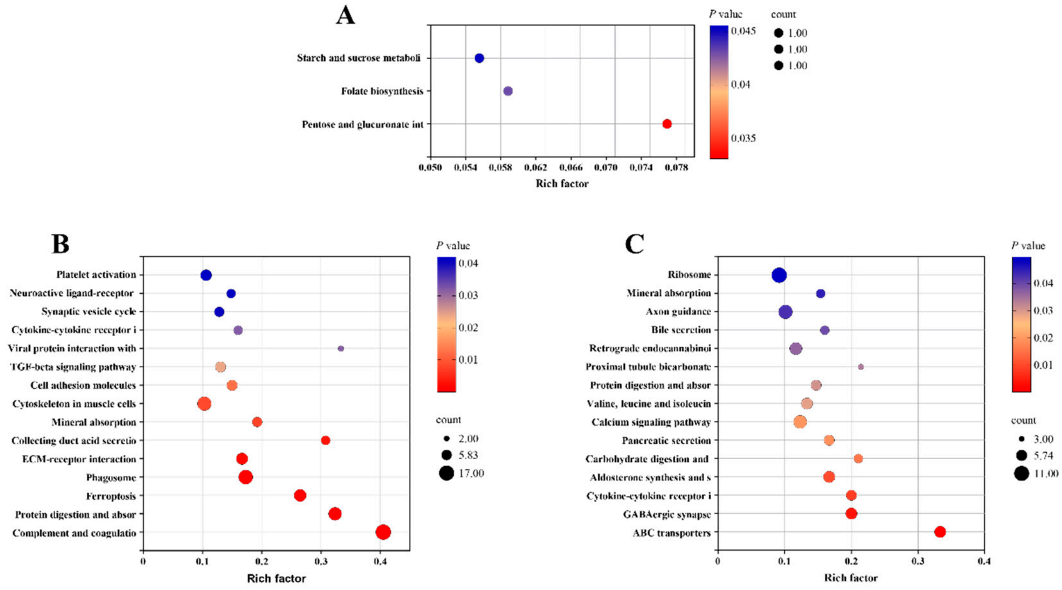


**Figure S2.** KEGG Pathway Enrichment Analysis of Differentially Expressed Proteins (DEPs) in Neuro-2a Cells Exposed to DTX1, AZA3, and YTX. (**A**) DTX1 treatment group; (**B**) AZA3 treatment group; (**C**) YTX treatment group. Bubble size indicates the number of DEPs mapped to each pathway, and the bubble color represents the enrichment significance (−$\log_{10}$ *p*-value). Rich factor is defined as the ratio of DEPs mapped to a pathway to the total number of annotated proteins in that pathway.

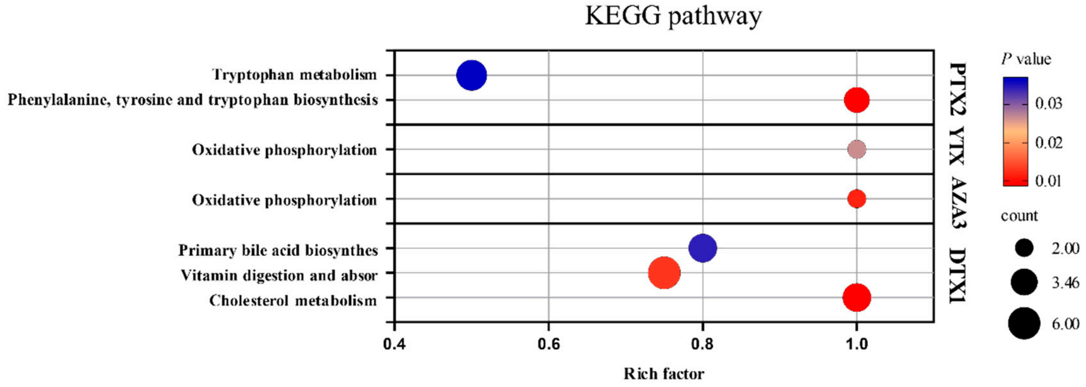


**Figure S3.** KEGG pathway enrichment analysis of differentially expressed metabolites (DEMs) in Neuro-2a cells exposed to DTX1, AZA3, YTX, and PTX2. Bubble size indicates the number of DEMs mapped to each pathway, and the bubble color represents the enrichment significance (−log$_{10}$ *p*-value). Rich factor is defined as the ratio of DEMs mapped to a pathway to the total number of annotated metabolites in that pathway.

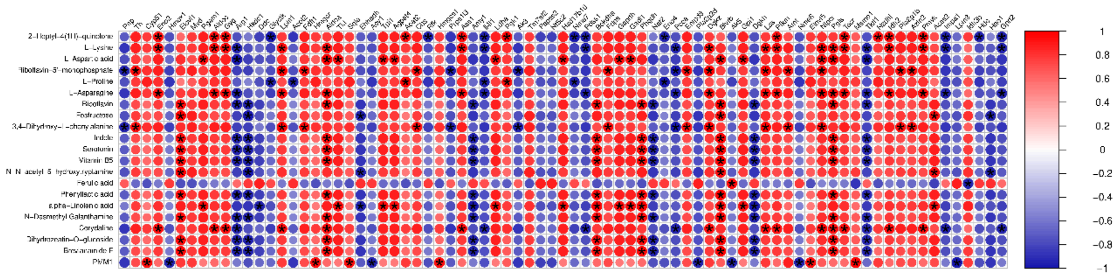

**Figure S4.** Complete Spearman correlation heatmap of all DEGs with DEPs or DEMs in biosynthesis of secondary metabolites in DTX1-treated Neuro-2a cells. Red and blue colors indicate positive and negative correlations, respectively. Spearman correlation coefficients were calculated for pathway-related DEGs and corresponding DEPs or DEMs, and p values were adjusted for multiple testing using the Benjamini–Hochberg false discovery rate (FDR) method. PMM1, phosphomannomutase 1 (* FDR-adjusted $p < 0.05$).

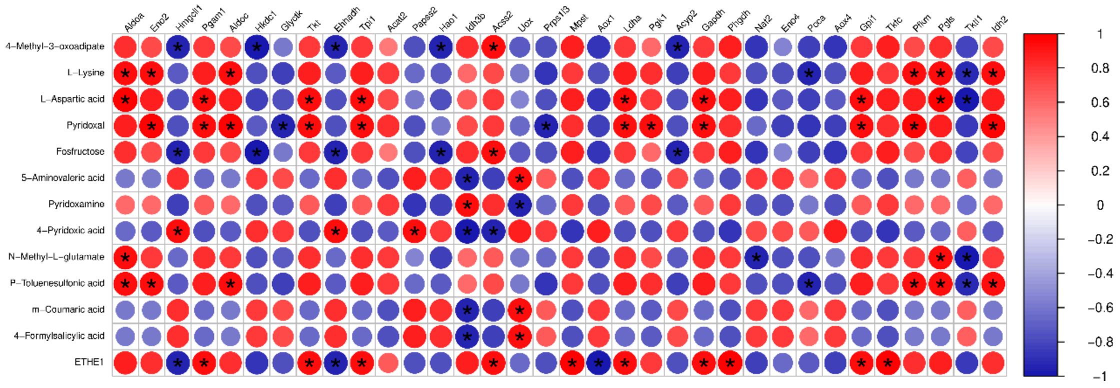

**Figure S5.** Complete Spearman correlation heatmap of all DEGs with DEPs or DEMs in microbial metabolism in diverse environments in DTX1-treated Neuro-2a cells. Red and blue colors indicate positive and negative correlations, respectively. Spearman correlation coefficients were calculated for pathway-related DEGs and corresponding DEPs or DEMs, and p values were adjusted for multiple testing using the Benjamini–Hochberg false discovery rate (FDR) method. ETHE1, persulfide dioxygenase ETHE1, mitochondrial (* FDR-adjusted $p < 0.05$).

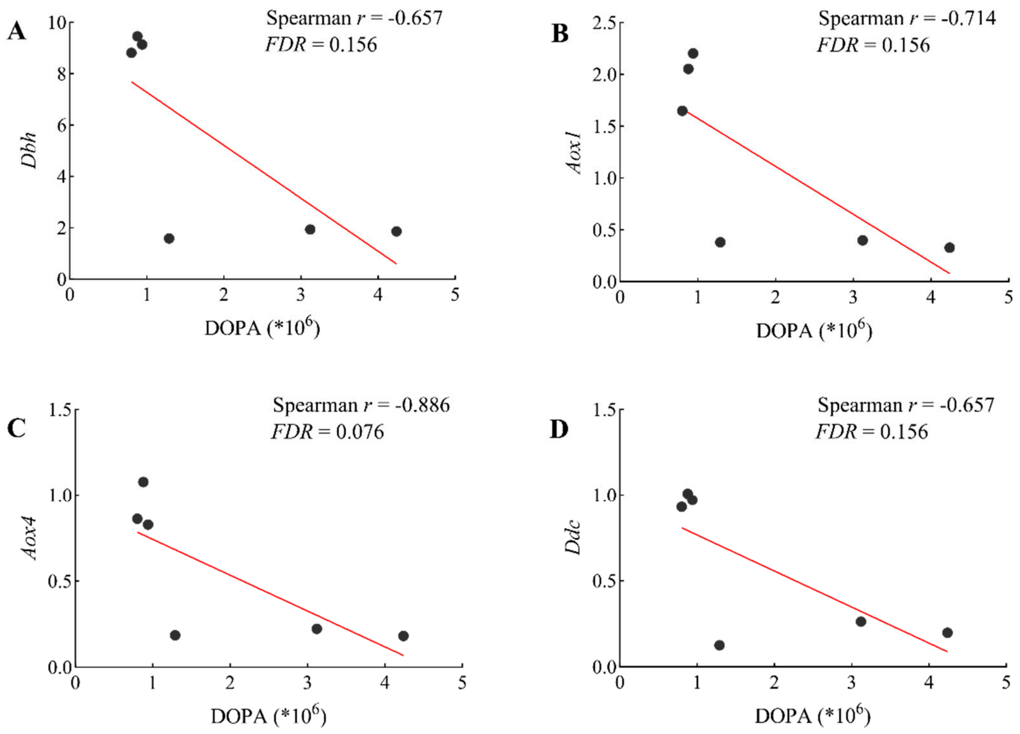


**Figure S6.** Spearman correlation scatter plot between all DEGs and DEM in Tyrosine metabolism (PTX2-treated Neuro-2a cells). Correlations between DOPA abundance and the expression levels of *Dbh* (**A**), *Aox1* (**B**), *Aox4* (**C**), and *Ddc* (**D**). Each dot represents an individual sample, and the red line indicates the fitted trend. Spearman correlation coefficients ($r$) and $p$ values are indicated in each panel. The $p$ values were adjusted for multiple testing using the Benjamini–Hochberg false discovery rate (FDR) method. DOPA, 3,4-Dihydroxy-L-phenylalanine.
